# The Dermatology Life Quality Index (DLQI) used as the benchmark in validation of 101 quality‐of‐life instruments: A systematic review

**DOI:** 10.1111/jdv.20321

**Published:** 2024-09-13

**Authors:** J. Vyas, J. R. Johns, Y. Abdelrazik, F. M. Ali, J. R. Ingram, S. Salek, A. Y. Finlay

**Affiliations:** ^1^ Centre for Medical Education, School of Medicine Cardiff University Cardiff UK; ^2^ Division of Infection and Immunity, School of Medicine Cardiff University Cardiff UK; ^3^ University Hospitals Birmingham, NHS Foundation Trust Birmingham UK; ^4^ School of Life and Medical Sciences University of Hertfordshire Hatfield UK

## Abstract

**Background:**

The validation of psychometric measures requires use of other established and standardized validated measures. The Dermatology Life Quality Index (DLQI) is the most widely used tool to measure the burden of skin diseases and assess effectiveness of interventions based on patients' perspective.

**Objectives:**

The objective of this study was to systematically analyse peer‐reviewed publications describing use of the DLQI in validation of other patient‐reported outcome (PRO) and quality‐of‐life (QoL) measures.

**Methods:**

Seven databases were searched for papers published between January 1994 and December 2022 for articles containing data using DLQI in the validation of other PRO/QoL measures. The methodology followed PRISMA guidelines. The protocol was prospectively registered on PROSPERO.

**Results:**

Of 1717 screened publications, 122 articles including 30,727 patients from 34 different countries with 41 diseases met the inclusion criteria. The DLQI was used in validation of 101 measures: 80 dermatology‐specific QoL measures, mostly disease‐specific, and 21 generic measures. Of these studies, 47 were cross‐cultural adaptations, 116 single arm, 100 were cross‐sectional, 18 longitudinal and six randomized placebo controlled. DLQI was used for 14 known group, and correlation for 10 construct, 101 convergent, 10 concurrent, 10 divergent/discriminant and three criterion validity tests using Mann–Whitney (2), Spearman's (80), Pearson's correlation (26) and Student's *t*‐test (1). The DLQI was used in responsiveness analysis in 13 studies.

**Conclusions:**

This review identified widespread use of the DLQI in validation of other dermatology PRO/QoL measures and confirmed the central role that the DLQI plays as a benchmark in instrument development and validation across dermatology and beyond. The use of the DLQI by so many developers of other instruments has provided a common standard for comparability.

## Graphical abstract

This study systematically analysed peer‐reviewed publications describing use of the DLQI in validation of other PRO and QoL measures and identified widespread use of the DLQI as a benchmark in validation of other dermatology PRO/QoL measures. It confirms the central role DLQI plays in the development of novel instruments and validation across dermatology and beyond.

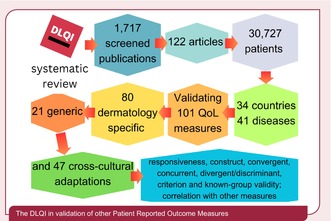

**Key points**

**Why was the study undertaken?**

The validation of psychometric measures requires use of other established and standardized validated measures. This study therefore systematically analysed peer‐reviewed publications describing use of the DLQI in validation of other PRO and QoL measures.

**What does this study add?**

This review identified widespread use of the DLQI as a benchmark in validation of other dermatology PRO/QoL measures and confirmed the central role DLQI plays in the development of novel instruments and validation across dermatology and beyond.

**What are the implications of this study for disease understanding and/or clinical care?**

Use of the DLQI by so many developers of other instruments has provided a common standard for validation. Developers of further novel PROMs for dermatology may thus consider including the DLQI as a comparator when planning their construct validation studies.

## INTRODUCTION

The validation of a quality of life (QoL)^1^, ^2^ instrument enhances its credibility and the quality of the research it supports. Validating novel QoL instruments against an established standard measure is a critical part of the process of psychometric testing^3^, ^4^ to give confidence of their use in both research settings and routine clinical practice. It is important that researchers and clinicians have access to information concerning the validation of an instrument.

There are many dermatology instruments designed for clinical scoring of disease severity. Over the last three decades, many instruments have also been designed to measure the impact of skin disease on quality of life, as well as to measure general or specific aspects related to dermatological conditions, for example stigmatization, social anxiety or depression. Instruments scored by patients lead to patient‐reported outcomes (PROs)^5^ that is ‘a report of the status of a patient's health condition that comes directly from the patient without interpretation of the patient's response by a clinician or anyone else’. Many aspects of patients' experiences with illness, medication and health care are best captured by PROs.^6^ In dermatology these measures, as part of establishing construct validity, should be compared with a well‐established measure that assesses closely related constructs. The wide adoption by researchers of the same comparator may add some degree of uniformity to this aspect of validation.

The aim of this study was to systematically analyse peer‐reviewed publications for the use of the Dermatology Life Quality Index (DLQI) in the validation of other PRO and QoL measures. The study includes both general and disease‐specific dermatological measures, and other general QoL measures.

## METHODS

### Scope

For this study, we defined validation as the collection and analysis of data to assess the validity and reliability of a QoL instrument to determine the extent to which an instrument measures what it purports to measure,^3^, ^4^ and defined PRO measures as those completed directly by the patient based on their own perception, including QoL; patient satisfaction; and/or signs and symptoms.

Our eligibility criteria for use of the DLQI in validation of other PRO‐QoL measures included studies that presented data and analysis of other PRO‐QoL measures that used the DLQI were the following:

- for determining subgroups in known‐group analysis,
- as an anchor in responsiveness and
- in correlation with other QoL/PRO measures.

Ineligible criteria for inclusion are the following:

- Correlations with non‐patient (e.g. physician) reported measures (mostly severity indices), for example PASI or PROs that were not QoL measures.

### Data sources

This study follows 2020 PRISMA guidelines for reporting systematic reviews.^7^ The study protocol and full search strategy was published on PROSPERO Prospective Register of Systematic Reviews (CRD42022308453).^8^ Medline (Ovid), Cochrane Library, EMBASE, Web of Science, SCOPUS, CINAHL(EBSCO) and PsycINFO online databases from 1 January 1994 (DLQI creation) to 31 December 2022 were searched independently by two authors (JJ and JV), and results corroborated. Search terms included ‘DLQI’ and ‘dermatology life quality index’. As complete a list as possible of validation search terms was used to ensure comprehensive coverage without creating excessive non‐relevant data. Database‐specific ‘article type/study type’ keywords and language keywords (English) were also used to search the required types of study to be included. Because of the difficulty of age selection (16 years old and over) using database search terms, all ages were included in the search, and those below the inclusion age were filtered manually in EndNote. Duplicate records were excluded. Search filtering by online databases, for example for the English language only, were not always complete, and some articles were further excluded during the data extraction process.

### Search strategy/selection

A set of eligibility criteria were applied for selection of the included studies (Table 1). Search results were imported into EndNote20®.^9^ Three authors (JJ, JV and YA) independently compared study titles and abstracts retrieved by searches against the inclusion and exclusion criteria and examined full study texts. Rejected studies were recorded with reasoning. A fourth author (SS) resolved and recorded any study selection disagreements.^7^

**TABLE 1:** Eligibility criteria for study selection.

Variable	Inclusion	Exclusion
Patients	Any gender, ethnicity, settings, countries Any inflammatory and non‐inflammatory dermatological conditions	Persons under the age of 16 (the DLQI was originally designed and validated for use with subjects aged 16 years and older)
Methods	Adaptive clinical trial, case reports, clinical study, clinical trial, controlled clinical trial, equivalence trial, evaluation study, multicentre study, observational study, randomized controlled trial, validation study Published between 1 January 1994 and 31 December 2022	Not in English language ‘Grey’ literature including dissertations, conference abstracts, reports, editorials, letters to editors, commentaries, protocols, reviews, conference proceedings and dissertations
Outcomes	Study presented at least one element of validation using the DLQI	DLQI not used in validation

### Data extracted

The recorded Information included the study aim, disease studied, disease severity, number of sites, study countries, the number of subjects for which DLQI data were collected, the study type and design, the name of the instrument(s) being validated, and details of validation methods used including type, statistical test or specific analysis methods, for example known group, construct validity, responsiveness or receiver operating characteristic (ROC). Data on cross‐cultural adaptations were also collected. Known‐group analysis was captured when statistical testing was applied to defined groups where there would be an expected difference, for example disease severity with the DLQI as the anchor or outcome as the test variable.

### Data extraction and synthesis

For data extraction, guidance of the Cochrane Handbook for Systematic Reviews of Interventions was followed.^10^ A REDCap database^11^, ^12^, ^13^ (a secure web application for building/managing online surveys and databases) was created. The authors JJ, JV and YA independently extracted data from the included publications to parallel REDCap database tables, and an adjudicator (SS) resolved any disagreements in data extraction. Missing data were noted in the data templates, but none was sufficiently important to contact original authors. Racial bias in research can impact a study's validity, reliability and relevancy.^14^, ^15^, ^16^ Minoritized populations have different outcomes, in part due to genetic ancestry.^17^ Recruiting for diversity is therefore essential and results should be stratified by race/ethnicity if relevant to the study,^18^ but this aspect is rarely addressed in systematic reviews. Appraisal of representation of minority ethnic participants in the studies was conducted using Naicker's Critically Appraising for Antiracism Tool.^19^

## RESULTS

**FIGURE 1:**
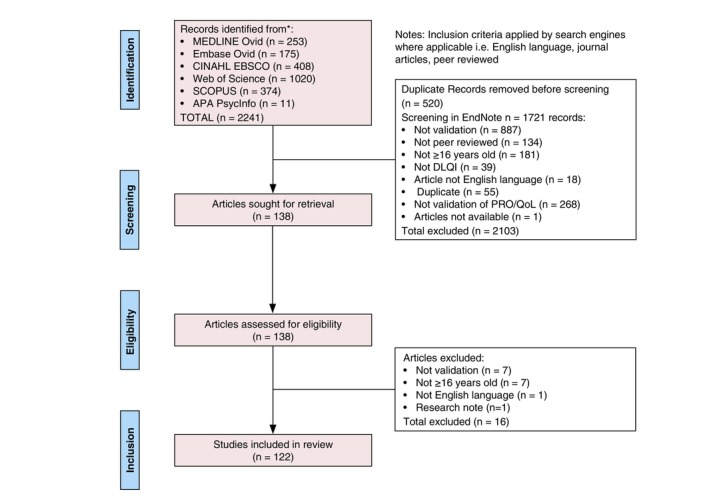
PRISMA flow diagram reporting the number of records identified from each database or searched and the number of articles matching the criteria that were included in the study.

**FIGURE 2:**
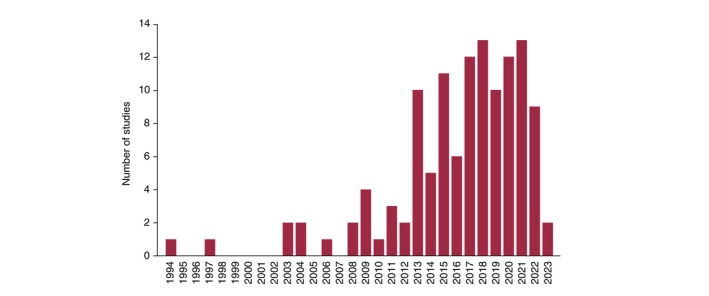
Number of Dermatology Life Quality Index (DLQI) studies published in peer‐reviewed journals view by year.

A total of 1721 studies resulted from database searching, after removing 520 duplicates. After filtering these in an EndNote^9^ database for inclusion/exclusion criteria, 138 full‐text articles were assessed, of which 122, describing research on 30,727 patients, met the inclusion eligibility criteria (Figure 1). Publications of validations using the DLQI are increasing, with the majority (76%) reported in the last 10 years (Figure 2).

### Study sites, settings and types

Seventy‐one (58.2%) of the studies were conducted at a single site, 46 (37.7%) were multicentre, and 5 (4.1%) were not specified. Of the multicentre studies, 31 (25.4%) were conducted at more than two sites. Most studies (116, 95.1%) did not involve any intervention and were not part of a clinical trial. The original study designs also comprised six randomized placebo controlled trials (RCT).

The study designs used, from which validation analysis was also gained, comprised 116 single arm (a single routine treatment or all treatments analysed together), six multiple arm, 100 cross‐sectional, 18 longitudinal, one parallel group, one comparator controlled, two Phase II RCT and four Phase III RCT studies (some of which fell under multiple categories described here).

Studies were conducted in at least 34 different countries: Two reported multiple countries without listing details, and two did not report the location of the study. The main countries where studies were conducted are given in Table 2.

**TABLE 2:** Main countries where studies were conducted.

Country	Number of studies (% of total)
United States	22 (18.0)
France	14 (11.5)
Germany	14 (11.5)
Brazil	13 (10.7)
Italy	10 (8.2)
China	9 (7.4)
Hungary	9 (7.4)
Poland	9 (7.4)
United Kingdom	9 (7.4)
Spain	7 (5.7)
Denmark	7 (5.7)
Australia	7 (5.7)
Multiple	23 (18.9)
Countries <5 studies	22 (18.0)
Countries with only 1 study carried out	10 (8.2%)

Fifty studies (41.0%) did not specify explicitly which language version of the DLQI was used, while five studies (4.1%) used multiple language versions. The Brazilian Portuguese (13, 10.7%) version was the most reported followed by the original English version of the DLQI (12 studies, 9.8%), Chinese Mandarin (5, 4.1%), Hungarian (4, 3.3%), Turkish (4, 3.3%), Chinese simplified (3, 2.5%), German (3, 2.5%) and Spanish (3, 2.5%). At least 18 different language variants (including specific adaptations, e.g. Arabic for UAE) were used in the studies.

### Disease profile

The validation studies included 41 different diseases. The disease reported in the most studies was psoriasis (*n* = 22, 15.3%), followed by atopic dermatitis (*n* = 16, 11.1%), vitiligo (*n* = 11, 7.6%), urticaria (*n* = 11, 7.6%), hidradenitis suppurativa (*n* = 8, 5.6%) and acne (5, 3.5%). Overall, studies recruited patients with mild (*n* = 41, 33.6%), moderate (*n* = 45, 36.9%) and severe (*n* = 49, 40.2%) disease (some studies having more than one category), with 61 (50.0%) unspecified. The PRO/QoL instruments validated and associated diseases are given in Table S1.

### Responsiveness to change

Responsiveness to change,^20^ or sensitivity, refers to a measure's ability to detect meaningful changes in a construct over time or in response to an intervention or treatment. A measure with good responsiveness to change should be able to detect changes (improvement or deterioration) in the construct being measured that are significant and relevant to the individual or group being studied.^21^ Thirteen studies of PRO measures demonstrated score change in patients' QoL before and after treatment, using the DLQI as an anchor to assess this responsiveness to change (Table 3). Responsiveness^20^, ^21^ was determined using a number of methods including Cohen's *d* effect size,^22^, ^23^ Spearman and Pearson correlations.^24^

**TABLE 3:** Dermatology Life Quality Index (DLQI) used in analysis of responsiveness to change in PRO‐QoL instruments.

Author/date	PRO/QOL measure	Disease	Country	No. of patients	Effect size (ES)	DLQI scores change correlated with change in a clinical measure	Correlation of DLQI with another measure	Standardized response means (SRM)	Results	Other
Dauden 2012^25^	PSO‐LIFE	Psoriasis	Spain	260	+	+			Effect sizes for the DLQI = 0.44 (baseline and final visit mean scores). Changes in score on the PSO‐LIFE showed moderate to high correlations with changes in score on the DLQI (*r* = −0.69)	
Gabes 2021^26^	Hyperhidrosis Quality of Life Index (HidroQoL)	Hyperhidrosis	Germany, Austria, Poland, Hungary, United Kingdom, Sweden, Denmark	170			+		HidroQoL and DLQI correlation between total scores using Spearman's rank: 0.69 (*p* < 0.05). Also used DLQI as an anchor to calculate the MID using the anchor‐based approach and integrated approach which combines the anchor used and distribution‐based approaches	Correlations of the Hyperhidrosis Quality of Life Index (HidroQoL) total change score and the DLQI change score‐Spearman's Rank was used
Judson 2015^27^	Sarcoidosis Assessment Tool (SAT)	Sarcoidosis	United States	173	+		+		Known group: SAT Module Scores within DLQI Anchor at Week 16: Sarcoidosis–Skin Concerns: DLQI Total a. 0–1 (*n* = 17) ES = 0.63; DLQI Total b. 2–5 (*n* = 17) ED = 1.05; DLQI Total c. 6–10 (*n* = 11) ES = 0.56: DLQI Total d. 11–30 (*n* = 10) null. *p* < −0.001. Sarcoidosis–Skin Stigma: DLQI Total a. 0–1 (*n* = 17) ES = 1.24; DLQI Total b. 2–5 (*n* = 17) ED = 1.04; DLQI Total c. 6–10 (*n* = 11) ES = 0.86: DLQI Total d. 11–30 (*n* = 10) null. *p* < −0.001	Effect sizes (mean difference/pooled SD) were calculated for the differences between adjacent groups to allow for comparisons with other instruments. Change in SAT Component Scores from Baseline to Week 16 with DLQI: Satisfaction with Roles and Activities: DLQI worsened >3.2 DLQI (*n* = 4) ES = 0.17 *p* = 0.75, no change ±3.2 (*n* = 62) ES = 0.38 *p* = 0.004, DLQI improved >3.2 (*n* = 13) ES = 0.52 *p* = 0.084 Skin Concerns: DLQI worsened >3.2 DLQI (*n* = 4) ES = 0.78 *p* = 0.22, no change ±3.2 (*n* = 39) ES = −0.42 *p* = 0.012, DLQI improved >3.2 (*n* = 12) ES = −1.00, *p* = 0.005 Skin Stigma: DLQI worsened >3.2 DLQI (*n* = 4) ES = 0.52 *p* = 0.38, no change ±3.2 (*n* = 39) ES = −0.20 *p* = 0.22, DLQI improved >3.2 (*n* = 12) ES = −0.44, *p* = 0.16
Muhleisen 2009^28^	Pictorial representation of illness and self‐measure (PRISM)	Dermatitis (*n* = 71), Psoriasis (*n* = 36), Leg ulcer (*n* = 28), Tumour (*n* = 26)	Switzerland	227	+				PRISM showed the highest sensitivity to change between admission and discharge with PRISM showing an effect size of *d* = 0.54 (mean 46.47 SD 25.57 *p* < 0.001) compared with *d* = 0.54 (mean 32.75 SD 25.65 *p* < 0.001) for DLQI	Cohen's *d*
Oosterhaven 2020^29^	Quality of Life in Hand Eczema Questionnaire (QOLHEQ) Dutch	Eczema/Hand eczema	Netherlands	300		+			Responsiveness (change‐score validity) in changed patients between T0 (baseline) and T2 4–12 weeks. Pearson's correlation	Change QOLHEQ – GRC > Change DLQI – GRC correlation 0.46 vs. 0.38. Change QOLHEQ – change Photoguide (physician) > Change DLQI – change Photoguide (physician) corr. 0.46 vs. 0.45. Change QOLHEQ – change HECSI > Change DLQI – change HECSI corr. 0.40 vs. 0.35. Change QOLHEQ – Change DLQI *r* = 0.56 (*N* = 124, patients that had changed at T2 according to the GRC scale). Hypothesis confirmed
Puelles 2022^30^	9SD‐NRS (Sleep Disturbance Numerical Rating Scale)	Atopic dermatitis	United States, Australia, Canada, Germany, Poland, France	207		+			Correlation moderate between the change in SD NRS and change in DLQI total score baseline to Week 24 (*r* = 0.41 *p* < 0.001)	Spearman's correlation
Schwartzman 2021^31^	Patient‐Reported Outcomes Measurement Information System Global Health (PGH)	Atopic dermatitis	United States	994		+			Correlation of change in DLQI and PGH T score changes from baseline to follow‐up: PGH‐P4 T score −0.46, PGH‐M4 T score −0.32, PGH‐P2 T score −0.36 PGH‐M2 T score −0.24; all *p* < 0.001	Spearman's correlation
Silverberg 2020^32^	PROMIS Itch Questionnaire Mood and Sleep (PIQ‐MS)	Atopic dermatitis	United States	410		+			Change in PIQ itch severity: Spearman's correlations with DLQI – Numerical Rating Scale (NRS) worse 0.26, NRS average 0.33, verbal rating scale (VRS) worse 0.27, VRS average 0.28, all *p* < 0.001	Follow‐up visit duration of 0.3 ± 0.4 years (maximum 1.9 years) *N* = 374. Change in numeric rating scales (NRS) and verbal rating scales (VRS) vs. change in DLQI
Silverberg 2021^33^	Patient Health Questionnaire‐9 (PHQ9), Abridged version PHQ‐2	Atopic dermatitis	United States	458		+			Spearman's correlation: Change in DLQI with change in PHQ9 (*r* = 0.42) and PHQ‐2 (*r* = 0.33), *p* < 0.001 for all, *N* = 434	
Simpson 2019^34^	Atopic Dermatitis Control Tool (ADCT)	Atopic dermatitis	United States	2584		+			Change in ADCT total score vs. change in DLQI bands Spearman (95% CI): Month 1 vs. Baseline, *n* = 538 0.47 (0.40, 0.54); Month 2 vs. Baseline, *n* = 458 0.48 (0.41, 0.55); Month 3 vs. Baseline, *n* = 372 0.51 (0.43, 0.58); Month 6 vs. Baseline, *n* = 206 0.50 (0.39, 0.59); all *p* < 0.001	Responsiveness was evaluated using correlations between the change from baseline (to Months 1, 2, 3 and 6) in ADCT total score and the change from baseline in DLQI total score (Pearson product–moment). The same analysis was conducted using (Spearman's rank order correlation) (*r* ≥ 0.5) for DLQI bands and PGAD scores
Strober 2016^35^	Psoriasis Symptom Diary	Psoriasis		820			+		PSD item vs. DLQI; 1. Itching 0.66, 3. Stinging 0.63, 5. Burning 0.66, 7. Pain/cracking 0.64, 9. Pain 0.63, 11. Scaling 0.65, 13. Notice – colour 0.62; all correlations significant *p* < 0.01	Correlation method not given. Responsiveness: (*N* = 666) correlations using change from baseline to week 12
Vinding 2014^36^	Skin Cancer Quality of Life questionnaire (SCQoL)	Non‐Melanoma Skin Cancer	Denmark	101	+	+		+	Baseline to follow‐up ES = 0.06 *p* = 0.66 SRM = 0.05. As expected, no statistically significant change in the DLQI was observed. This was confirmed by the SRM and ES, showing moderate effects for the domain emotion and the single global item	Cohen's effect size
Warren 2021^37^	Psoriasis Symptoms and Impacts Measure (P‐SIM)	Psoriasis	United States, Canada, Belgium, Germany, Italy, UK, Hungary, Poland, Russian Federation, Australia, Japan, Korea	1002		+			All P‐SIM changes from baseline to week 16 item scores were strongly correlated (Spearman's) with DLQI total score (*r* = 0.60 to 0.69 *n* = 698) and DLQI item 1 score (*n* = 696 *r* = 0.50 to 0.74)	Spearman's correlation

### Known group

Known‐group analysis is a type of construct validity that measures an instrument's ability to detect hypothesized differences among distinct (independent) groups. Groups are generally defined using another independent measure,^38^ for example DLQI score bands of Hongbo et al.^39^ Group differences are then determined using a statistical test. The effect size can also be determined.^40^

Table 4 shows studies where known‐group validity (i.e. a type of construct validity) was analysed utilizing the DLQI. We included studies where known‐group analysis was performed, even if the authors had not stated an a priori hypothesis. Six of the 14 studies reported effect sizes, while other statistical tests were also performed in the known‐group analyses (ANOVA, ANCOVA, Student's *t*‐test, Mann–Whitney *U*‐test, Kruskal–Wallis and logistic regression models) to discriminate between the studied groups to show statistical significance. Known‐groups validity evidence is essential to provide confidence in the construct and use of a measure over a wide variety of groups (e.g. disease severity, disease location and disease duration).

**TABLE 4:** Known‐group validity analysis using the Dermatology Life Quality Index (DLQI).

Author/date	PRO/QOL measure	Disease	Country	No. of patients	Notes	Results
Dreyfus 2013^41^	Ichthyosis‐specific measure of quality of life (IQoL‐32)	Ichthyosis	France	59	Global severity: mild/moderate/severe/very severe; clinical severity evaluated by six visual analogue scales evaluating the intensity of erythema, scales, pruritus, cutaneous pain, eye pain and daily life disability	Global severity IQoL‐32 score vs. DLQI score mean +/SD. Mild *n* = 11 10.09 +/1 6.89, Moderate *n* = 20 8.75 ± 3.96 Severe *n* = 15 16.33 ± 5.92, Very severe *n* = 8 20.25 ± 6.32. Severe vs. mild, *p* < 0.05, severe vs. moderate *p* < 0.05, very severe vs. mild *p* < 0.05, very severe vs. moderate *p* < 0.0001
Judson 2015^27^	Sarcoidosis Assessment Tool (SAT)	Sarcoidosis	United States	173	SAT Module Scores within DLQI Anchor at Week 16: Sarcoidosis–Skin Concerns DLQI Total a. 0–1 (*n* = 17) ES = 0.63; DLQI Total b. 2–5 (*n* = 17) ED = 1.05; DLQI Total c. 6–10 (*n* = 11) ES = 0.56: DLQI Total d. 11–30 (*n* = 10) null. *p* < −0.001. DLQI anchor groups as per Hongbo with DLQI with very large and extremely large groups merged: scores 11–30	SAT Module Scores within DLQI Anchor at Week 16: Sarcoidosis–Skin Stigma: DLQI Total a. 0–1 (*n* = 17) ES = 1.24; DLQI Total b. 2–5 (*n* = 17) ED = 1.04; DLQI Total c. 6–10 (*n* = 11) ES = 0.86; DLQI Total d. 11–30 (*n* = 10) null. *p* < −0.001
Kirby 2021^42^	Patient global assessment (PtGA) for hidradenitis suppurativa	Hidradenitis suppurativa	United States, Denmark	441	DLQI score bands. Based on complete case analysis, *n* = 432 for combined US and Danish responses. ANCOVA: PtGA responses as the dependent variable and adjusted for country, age or sex	PtGA Mean (SD): No effect *N* = 24 0.42 (0.65), small effect *N* = 89 0.90 (0.74), moderate effect *N* = 102 1.55 (0.77), very large effect *N* = 127 2.55 (1.00), extremely large effect *N* = 85 3.56 (0.75): *p* < 0.001
Kirby 2020^43^	Hidradenitis Suppurativa Quality of Life (HiSQOL)	Hidradenitis suppurativa	United States, Denmark	405	Known‐groups validity was evaluated as the differences in HiSQOL scores among known scoring bands for the DLQI (Hongbo) using ANOVA	DLQI score bands vs HiSQOL score mean ± SD. No effect *n* = 22 3.1 ± 6.2, small effect *n* = 83 9.6 ± 6.5, moderate effect *n* = 101 19.1 ± 7.6, very large effect *n* = 116 32.0 ± 9.5, extremely large effect *n* = 83 48.6 ± 8.4; *p* < 0.0001
Koszoru 2023^44^	EuroQol EQ5D 3L and 5L	Atopic dermatitis	Hungary	218	Kruskal–Wallis test and Effect size (ESs ≥0.01 as small, ≥0.06 as moderate and ≥0.14 as large). DLQI anchor used Hongbo banding	Known group: EQ‐5D‐3L *p* < 0.001 Es = 0.489; EQ‐5D‐5L *p* < 0.001 Es = 0.384 *p* < 0.001, with relative efficiency RE = 1.275. Both the 3L and 5L were able to distinguish across predefined groups of patients based on severity and skin specific HRQoL (i.e. DLQI score bands) with moderate to large effect sizes (0.080–0.489)
Kulthanan 2019^45^	Angioedema quality of life questionnaire (AE‐QoL)	Urticaria	Thailand	86	To find cut‐off values of the AE‐QoL questionnaire that differentiated patients with ‘no effect’ from patients with ‘moderate to large effect’ on health‐related quality of life (HRQoL) using the PGA‐QoL and DLQI, patients were classified into three groups using the DLQI and PGA‐QoL scores in this study, as follows: (i) ‘no effect’ (DLQI scores of 0–1, PGA‐QoL score of 0); (ii) ‘small effect’ (DLQI scores of 2–5, PGA‐QoL score of 1); and, (iii) ‘moderate to large effect’ (DLQI scores of 6–30, PGA‐QoL scores of 2–4)	Using Hongbo banding, AE‐QoL total value mean ± SD (median) was: DLQI no effect 11.6 ± 13.6 (5.9), small effect 26.0 ± 15.3 (24.3), moderate effect 39.7 ± 17.3 (38.2), very large effect 42.2 ± 19 (42.6), extremely large effect 0. AE‐QoL showed high known‐groups validity as it was able to discriminate among patients who showed differences in HRQoL impairment as assessed by the use of the PGA‐QoL and the DLQI banding
Kulthanan 2016^46^	Chronic Urticaria Quality of Life Questionnaire (CU‐Q2oL) Thai	Urticaria	Thailand	166	Kruskal–Wallis test: DLQI score bands (Hongbo) were used to differentiate five levels of HRQoL impairment: no effect (score of 0–1), small effect (score of 2–5), moderate effect (score of 6–10), large effect (score of 11–20) and extremely large effect (score of 21–30)	A statistically significant differences in the CU‐Q2oL total score among the and five DLQI groups were found (*p* < 0.0001)
Puelles 2022^30^	9SD‐NRS (Sleep Disturbance Numerical Rating Scale)	Atopic dermatitis	United States, Australia, Canada, Germany, Poland, France	207	Comparison of mean SD NRS average weekly scores at baseline by DLQI total score bands (Hongbo) using ANOVA adjusted for multiple comparisons based on the Scheffe method. Known‐groups validity was not supported when using clinician‐reported scales such as IGA (*p* = 0.25) or EASI (*p* = 0.11)	DLQI (*N*, total score (SD)) 0–1 (no effect) *N* = 0; 2–10 (small‐to moderate effect) *N* = 47 7.2 (1.8); 11–20 (very large effect) *N* = 100 7.7 (1.6); 21–30 (extremely large effect) *N* = 60 8.7 (1.1); test value 14.5, *p* < 0.001. The SD NRS was able to discriminate participants in the expected direction according to groups defined by DLQI total score at baseline (*p* < 0.001)
Rencz 2022^47^	ICECAP‐A	Multiple	Hungary	618		ICECAP‐A index score Mean (SD), Median (IQR): DLQI ≤ 10 *n* = 552 0.70 (0.19), 0.75 (0.25); DLQI > 10 *n* = 66 0.61 (0.23), 0.62 (0.37). *p* < 0.002
Rentz 2020^48^	Psoriasis Symptom Scale (PSS)	Psoriasis	Australia, Austria, Belgium, Canada, Czech Republic, France, Germany, Japan, Mexico, Poland, Portugal, South Korea, Spain, United States	1000	To evaluate known‐groups validity, the PSS total scores were examined using an analysis of covariance (ANCOVA) model at weeks 12 and 16. Groups for these ANCOVAs were defined as DLQI total scores of 0–1 or ≥2	Evidence of known‐groups validity (ANCOVA) was shown with a significant association between the PSS total score and two‐category DLQI scores (0–1, ≥2) at weeks 12 and 16 (*p* < 0.0001)
Sampogna 2015^49^	Psodisk	Psoriasis	Italy	320	Comparison between PSOdisk categories (<9, 9–15, 16–30, 31–50, >50) and DLQI Hongbo categories (0–1, 2–5, 6–10, 11–20, 21–30) at baseline	
Schwartzman 2021^31^	Patient‐Reported Outcomes Measurement Information System Global Health (PGH)	Atopic dermatitis	United States	994	None of the PGH or DLQI scores were able to differentiate between the lowest three levels of atopic dermatitis severity. However, DLQI was slightly better at distinguishing between different levels of self‐reported disease	There were significant and stepwise increases of PGH‐P4, PGH‐M4, PGH‐P2 and PGH‐M2 T scores and EQ5D at each level of severity for self‐reported global severity. T scores and DLQI scores showed similar multilevel area under the curve (AUC), indicating poor known‐groups validity in predicting self‐reported global atopic dermatitis severity overall
Simpson 2019^34^	Atopic Dermatitis Control Tool (ADCT)	Atopic dermatitis	United States	2584	Known group (Mann–Whitney *U*‐test or *t*‐test): mean ADCT total scores (baseline, months 1, 2, 3, 6) were compared across adjacent subgroups of patients based on categories of DLQI responses: no effect on patient life (score range: 0–1)	
Yfantopoulos 2017^50^	EQ5D 3L and 5L	Psoriasis	Greece	396	Known‐group DLQI levels (Hongbo): No effect (*N* = 64), Small effect (*N* = 160), Moderate effect (*N* = 83), Very large effect (*N* = 76), Extremely large effect (*N* = 10)	EQ5D‐3L Mean (SD) No effect 0.86 (0.14), Small effect 0.75 (0.23), Moderate effect 0.69 (0.25), Very large effect 0.63 (0.29), Extremely large effect 0.64 (0.33). EQ5D‐5L Mean (SD) No effect 0.85 (0.14), Small effect 0.76 (0.22), Moderate effect 0.72 (0.23), Very large effect 0.64 (0.26), Extremely large effect 0.64 (0.32). EQ‐5D‐3L and EQ‐5D‐5L both *p* < 0.001. RE: relative efficiency one‐way ANOVA *F* statistics (F5L/F3L) RE = 0.87

*Note*: Hongbo refers to Hongbo et al.^39^

### Studies assessing the correlation of PRO/QoL instruments with the DLQI

In this systematic review, we identified correlations of the DLQI with other PRO/QOL instruments when used to test their construct validity, convergent validity, concurrent validity, divergent validity and criterion validity (Table 5). Correlations with non‐PRO or non‐QoL measures, for example severity scales were not included. Of 119 studies that published correlations, almost all were Spearman's (80) or Pearson's (26) tests with one Wilcoxon test, and 17 studies did not specify the testing method.

**TABLE 5:** Correlations of patient‐reported outcomes (PRO)/quality‐of‐life (QoL) instruments with the Dermatology Life Quality Index (DLQI).

Author/date	PRO/QOL measure	Disease	Country	No. of patients	Construct	Convergent: Correlation with…	Concurrent	Divergent/discriminant	Criterion	Spearman's	Pearson's	Results	Validity notes
Alarcon 2017^51^	Actinic Keratosis Quality of Life questionnaire	Actinic keratosis	Spain	100		+				+		AKQoL showed high Spearman rank correlation with DLQI: total score *r* = 0.87, function *r* = 0.75, emotions *r*‐0.78, control *r* = 0.75, global item *r* = 0.76	
Aminizadeh 2022^52^	Skindex‐29	Any skin disease	Iran	200		+				+		*r* = 0.719, subscales Skindex‐29, *r* from 0.24 to 0.71 (*p* < 0.01)	
Arents 2019^53^	Atopic Eczema Score of Emotional Consequences (AESEC), Hospital Anxiety Depression Scale (HADS)	Atopic dermatitis	Multiple	1189		+				+		AESEC (0.546, *p* < 0.001, 95% CI = 0.505, 0.585), HADS‐D7 (ρ = 0.461 *p* < 0.001, 95% CI = 0.414, 0.505)	
Augustin 2004^54^	Freiburg Life Quality Assessment (FLQA)	Psoriasis and Psoriatic arthritis	Germany	895		+					+	FLQA‐c domains and DLQI showed significant correlations between 0.33 and 0.65 (all <0.01)	
Balkrishnan 2003^55^	Melasma Quality of Life scale (MELASQOL)	Melasma	United States	102		+						Correlation of MELASQOL with DLQI *r* = 0.84, *p* < 0.05	
Baoqi 2017^56^	Autoimmune Bullous Disease Quality of Life (ABQOL)	Bullous disease	China	101		+		+				Convergent validity: *r* = 0.77, *p* < 0.001; Discriminant validity Fisher exact test, *p* = 0.236	Discriminant validity was assessed by comparing the proportions of insensitive items in the ABQOL and DLQI
Bato 2021^57^	EQ5D 3L and 5L	Hidradenitis Suppurativa	Hungary	200		+				+		Correlations EQ5D‐3L and 5L: Mobility 0.396, 0.426, Self‐care 0.409, 0.469, Usual activities 0.547, 0.541, Pain/discomfort 0.628, 0.671, Anxiety/depression 0.564, 0.560, index score −0.722, −0.697, all *p* < 0.05	
Boleira 2014^58^	Psychosomatic Scale for Atopic Dermatitis	Atopic dermatitis	Brazil	44		+					+	Correlation between mean total DLQI scores and PSS‐AD was statistically significant (*r* = 0.78, *p* < 0.00001). Dimension II of PSS‐AD (maladjustment/relationships) correlated with all DLQI items. Dimension I (stress/laziness/insecurity) had moderate correlation with majority of DLQI items, particularly with items 1 (itching) (*r* = 0.42), 6 (sports) (*r* = 0.47) and 10 (treatment) (*r* = 0.44, *p* < 0.05)	
Bolton 2021^59^	Acceptance of Illness Scale and Person‐centred Dermatology Self‐care Index (PeDeSI)	Psoriasis	Egypt	116		+				+		There was a significant negative moderate correlation between the total scores of the AIS and DLQI questionnaires (Spearman ρ = −0.44, *p* < 0.01). A significant weak correlation was found between the total score of the PeDeSI and DLQI questionnaires (Spearman ρ = −0.23, *p* = 0.02)	
Boza 2015^60^	VitiQoL‐PB	Vitiligo	Brazil	74		+					+	Significant correlation between VitiQoL and DLQI (*r* = 0.776, *p* < 0.001). Good correlation between the total DLQI and subjects' assessment of the severity of their disease (*r* = 0.673, *p* < 0.001)	
Brzoza 2011^61^	Chronic Urticaria Quality of Life Questionnaire (CU‐Q2oL) Polish	Urticaria	Poland	126		+				+		Significant moderate and strong correlations between CU‐Q2oL subscales and respective DLQI items (0–45 to 0.67, *p* < 0.01) and domains. CU‐Q2oL subscale: Itching and DLQI item 1 *r* = 0.67, *p* < 0.001; Functioning and mean DLQI items 3, 5, 6, 7 *r* = 0.64, *p* < 0.001; Eating/Limits and DLQI item 4 *r* = 0.56, *p* < 0.001; Embarrassment and DLQI item 2 *r* = 0.45, *p* = 0.003	
Burdon‐Jones 2013^62^	Skin Cancer Quality of Life Impact Tool (SCQOLIT)	Malignant melanoma (MM) or non‐melanoma skin cancer (NMSC)	United Kingdom	110		+						The median (IQR, range) DLQI scores for the MM group was 2 (4, 0–13), for the NMSC group 1 (1, 0–4) and for the combined group 1 (2, 0–13). There was a significant difference between the SCQOLIT and DLQI scores (*p* < 0.001), and the same trend was seen for both the MM patients (*n* = 54, *p* < 0.001) and NMSC patients (*n* = 56; *p* < 0.001)	Wilcoxon signed‐rank test. Two groups: non‐metastatic (MM) and non‐metastatic skin cancer (NMSC)
Carcano 2018^63^	Skindex‐16	Atopic, seborrheic, dyshidrotic and contact eczema (*n* = 15), non‐melanoma skin cancer (*n* = 12), leprosy (*n* = 11), melasma (*n* = 11), acne (*n* = 8) and senile freckle (*n* = 7)	Brazil	110		+				+		All three Skindex‐16 scales exhibited strong correlation with DLQI scores (ρ = 0.664, 0.766 and 0.712 for the domains symptoms, emotions and functioning, respectively)	
Carroll 2008^64^	Dermatomyositis Skin Severity Index (DSSI)	Dermatomyositis	United States	98	+					+		DSSI and DLQI at WF study site: 0.38 (*p* < 0.0013); nonsignificant at 0.36 (*p* = 0.07) at UL study site	
Catucci Boza 2017^65^	VitiQoL‐PB	Vitiligo	Brazil	93		+				+		A strong correlation between the scores of the total VitiQoL and DLQI was observed (*r* = 0.81; *p* < Leisure (Q5, Q6) *R*s = 0.001); Symptoms and feelings (Q1, Q2) *R*s = 0.76 (<0.001); 0.84 (<0.001); Work and school (Q7) *R*s = 0.39 (<0.001); Personal relationships (*Q8, Q9) Rs = 0.66 (<0.001); Daily activities (Q3, Q4) *R*s = 0.76 (<0.001); Treatment (Q10) *R*s = 0.36 (<0.001)	
Cestari 2016^66^	Quality of life evaluation in epidermolysis bullosa (QoLEB) Brazilian Portuguese	Epidermolysis bullosa	Brazil	17		+					+	Strong correlations were found between scores on the QoLEB‐BP and DLQI (Pearson's *r* = 0.688, *p* = 0.002)	
Chen 2018^67^	Autoimmune Bullous Disease Quality of Life (ABQOL)	Bullous disease	China	86		+				+		*r* = 0.664, *p* < 0.001	
Cohen 2016^68^	Inverse Psoriasis Burden of Disease (IPBOD)	Psoriasis	United States	16		+				+		IPBOD VAS and DLQI *r* = 0.650 *p* = 0.006; IP‐BOD VAS and DLQI *r* = 602 *p* = 0.014. There was no correlation between the maximum overall scores of the two questionnaires (*r* = 0.361 *p* = 0.169)	
Corazza 2020^69^	Pictorial representation of illness and self‐measure (PRISM)	Lichen sclerosus (LS), lichen planus (LP), lichen simplex chronicus (LSC)	Italy	87		+				+		Moderately coherent results were seen between PRISM and DLQI (*q* = 0.5455, *p* < 0.001)	
Cozzani 2018^70^	PSOdisk	Psoriasis	Italy	50		+					+	There was a moderate significant correlation between PSOdisk and PASI (*r* = 0.62; *p* < 0.0001)	
Dauden 2012^25^	PSO‐LIFE	Psoriasis	Spain	260		+		+		+		The correlation with the DLQI global score was *r* = −0.76 (*p* < 0.01). The highest correlations with the DLQI were observed on the symptoms and perceptions dimension (*r* = −0.78, *p* < 0.01), followed by activities of daily living and leisure (*r* = −0.66, *p* < 0.01). The lowest correlation was seen with the DLQI work and study dimension (*r* = −0.40, *p* < 0.01)	Discriminant validity: standardized differences in PSO‐LIFE scores between patients with active and inactive disease were larger than the differences found on the DLQI and PDI (standardized differences of 0.85 standard deviations on the PSO‐LIFE compared with 0.79 on the DLQI and 0.62 on the PDI)
Deng 2018^71^	Rosacea‐specific Quality‐of‐Life instrument (RosQol)	Rosacea	China	265		+				+	+	*r* = 0.686 *p* < 0.01; Emotion *r* = 0.706, Symptoms *r* = 0.540, Function *r* = 0.361, all *p* < 0.01	Unclear which correlation statistic reported
Di Carlo 2017^72^	12‐item Psoriatic Arthritis Impact of Disease (PsAID‐12)	Psoriatic arthritis	Italy	144		+				+		Correlations between the comparable dimension of the PsAID Skin Score and the DLQI (ρ = 0.684, *p* < 0.0001)	
Dias 2011^73^	Chronic Urticaria Quality of Life Questionnaire (CU‐Q2oL) Brazilian Portuguese	Urticaria	Brazil	112		+					+	Mean DLQI and CU‐Q2oL scores: *r* = 0.76 *p* < 0.0001. Strong correlations with items 1 (itching/soreness/pain/stinging) (*r* = 0.67), 2 (embarrassment/self‐consciousness) (*r* = 0.69), 3 (shopping, home, garden) (*r* = 0.67), item 5 (social, leisure) (*r* = 0.70), item 7 (work, studying) (*r* = 0.62) and item 8 (social relationship) (*r* = 0.61) (*p* < 0.00001). CU‐Q2OL scale I (sleep/mental status/eating) was found to be more correlated with DLQI item 1 (*r* = 0.52), and scale III swelling/limits/look) was correlated with item 2 (*r* = 0.63) (*p* < 0.00001)	
Dimitrov 2019^74^	6‐item Stigmatization Scale and the Feelings of Stigmatization Questionnaire	Psoriasis	United Arab Emirates	39		+				+		Strong correlation between the total score of 6‐item Stigmatization Scale and DLQI (ρ = 0.54, *p* < 0.001). A significant negative moderate correlation was documented between the Feelings of Stigmatization Questionnaire and DLQI (ρ = −0.49, *p* = 0.001)	
Dreyfus 2013^41^	Ichthyosis‐specific measure of quality of life (IQoL‐32)	Ichthyosis	France	59		+				+		IQoL‐32 score was significantly positively correlated to DLQI score (*r* = 0.68, *p* < 0.0001)	
Eghlileb 2009^75^	Psoriasis Family Index‐15 (PFI‐15)	Psoriasis	United Kingdom	92		+				+		Family members' PFI‐15 scores and patients' DLQI score *r* = 0.54, *p* < 0.01	
Evenhamre 2017^76^	WHOQOL‐BREF	Pruritus, dermatitis, acne, psoriasis and rosacea	Sweden	198		+				+		*r* = 0.55, *p* < 0.01	
Ezzedine 2020^77^	Individual Burden of Psoriasis (I‐BOP)	Psoriasis	France	208		+					+	*r* = 0.77	
Ezzedine 2023^78^	Patient Unique Stigmatization Holistic tool in dermatology (PUSH‐D)	Atopic dermatitis/eczema, psoriasis, acne, rosacea, hair loss, skin ageing, visible facial scar, vitiligo	France	26		+					+	The 17‐item questionnaire correlated strongly with the DLQI 0.72 (*p* < 0.001)	
Ferreira 2019^79^	Chronic Urticaria Quality of Life Questionnaire (CU‐Q2oL) Portuguese	Urticaria	Portugal	162		+					+	Overall CU‐Q2oL and DLQI score *r* = 0.846, *p* < 0.001	Also compared criterion validity by comparing the dimensions of the CU‐Q2oL to the dimensions of the DLQI using Pearson's r correlation coefficients
Fotiou 2015^80^	Pictorial representation of illness and self‐measure (PRISM)	Psoriasis	Germany	108		+				+		Moderate correlation was observed between PRISM and DLQI (ρ = 0.45, *p* < 0.001). For patients with low disease activity PRISM and DLQI ρ = 0.62, *p* < 0.001	
Gabes 2021^26^	Hyperhidrosis Quality of Life Index (HidroQoL)	Hyperhidrosis	Germany, Austria, Poland, Hungary, United Kingdom, Sweden, Denmark	170		+				+		HidroQoL total score and DLQI *r* = 0.69, *p* < 0.05 (exact *p* value not stated but significant)	
Gergely 2020^81^	EQ‐5D‐5L, Skindex‐16	Hidradenitis suppuratvia	Hungary	200		+				+		Convergent validity (DLQI was compared with EQ‐5D‐5L, Skindex‐16 and DLQI‐R, MSS, PtGA VAS and HS‐PGA) with Spearman's rho: DLQI and EQ‐5D‐5L: *r* = 0.697 *p* < 0.05. DLQI and EQ VAS *r* = 0.512 *p* < 0.05. DLQI and DLQI‐R: *r* = 0.993, *p* < 0.05, DLQI and Skindex‐16 *r* = 0.859, *p* < 0.05. PtGA VAS and DLQI: *r* = 0.542, *p* < 0.05. HS‐PGA and DLQI: *r* = 0.418, *p* < 0.05. MSS and DLQI: *r* = 0.376, *p* < 0.05	Validity of EQ‐5D‐5L, Skindex‐16, DLQI and DLQI‐R
Gilet 2015^82^	REFlective evaLuation of psoriasis Efficacy of Treatment and Severity (REFLETS) QoL	Psoriasis	France	430		+					+	REFLETS scores were also moderately to highly correlated to DLQI scores (*r* = 0.36–0.82). Statistical method used not specified and *p* values not given	
Guo 2018^83^	CECA10 (Specific Questionnaire for Condylomata Acuminata) QOL	Condyloma acuminata	China	62		+				+		Moderate negative correlation (*r* = −0.50, *p* < 0.001) between the original CECA10 and DLQI	
He 2014^84^	Skindex‐16, Skindex‐29	Any skin disease	China	221		+				+		Moderate to good correlations between Skindex‐29 and DLQI, Skindex‐16 and DLQI (Skindex‐29, *r* from 0.43 to 0.84; Skindex‐16, *r* from 0.39 to 0.83)	
He 1012^85^	Psoriasis Disability Index (PDI) Chinese	Psoriasis	China	831		+				+		*r* = 0.78, *p* < 0.001, *N* = 815	
Heisterberg 2014^86^	Fragrance QoL instrument (FQL index)	Fragrance allergy	Denmark	550		+				+		DLQI with FQI Fragrance‐positive *r* = 0.7 *p* < 0.001, Non‐fragrance‐positive *r*‐0.74 *p* < 0.001, All respondents *r* = 0.72 *p* < 0.001; DLQI vs. Severity of eczema at this present moment (VAS), Fragrance‐positive respondents; *r* = 0.56, *p* < 0.001, Non‐Fragrance‐positive respondents; *r* = 0.60, *p* < 0.001	
Herd 1997^87^	Patient Generated Index (PGI)	Atopic dermatitis	United Kingdom	56		+			+			DLQI correlation with PGI was −0 to 52 (*p* < 0 to 001). Criterion validity DLQI vs PGI: Q1–0–36*. Q2–0–51**. Q3–0–39*, Q4–0–42**, Q5–0–40*, Q6–0–27, Q7–0–20, Q8–0–19, Q9–0–13, Q10–0–32; *p < 0–01; **p < 0–001	To assess criterion validity, the correlations between the PGI and DLQI, and between the PGI and individual questions of the DLQI; Construct validity was also measured by correlating results of the DLQI and PGI
Iwanowski 2021^88^	VITIQoL Polish	Vitiligo	Poland	97		+				+		Significant correlation between VitiQoL and DLQI (*r* = 0.90, *p* < 0.001). Good correlation between the total DLQI and subjects' assessment of the severity of their disease (*r* = 0.87, *p* < 0.001)	
Jorge 2021^89^	Skindex‐17 Brazil	Any skin disease	Brazil	217			+			+		Symptoms (ρ = 0.69, *p* < 0.01) and psychosocial conditions (ρ = 0.75, *p* > 0.01)	Symptoms dimensions of Skindex and the psychosocial dimension were compared with the DLQI
Judson 2015^27^	Sarcoidosis Assessment Tool (SAT)	Sarcoidosis	United States	173		+				+		Convergent criterion at Week 16 between SAT and DLQI: Satisfaction with Roles and Activities *r* = −0.22; Sarcoidosis‐Skin Concerns *r* = 0.66; Sarcoidosis‐Skin Stigma 0.69	
Kamudoni 2015^90^	HidroQoL	Hyperhidrosis	United States, Canada, Australia	595		+				+		HidroQoL correlated with the DLQI (*r*s = 0.60, *p* < 0.01)	
Kessel 2015^91^	Chronic Urticaria Quality of Life Questionnaire (CU‐Q2oL) Hebrew	Urticaria	Israel	119		+				+		CU‐Q2oL and DLQI score correlation: (*r* = 0.8, *p* < 0.01)	
Kirby 2020^92^	Severity and Area Score for Hidradenitis (SASH)	Hidradenitis suppurativa	United States	23				+		+		Correlation with the DLQI, showed a negative correlation of 0.41 [95% confidence interval (CI) −0.002 to 0.70], not statistically significant	
Kirby 2021^42^	Patient global assessment (PtGA) for hidradenitis suppurativa	Hidradenitis suppuratviva	United States, Denmark	441		+				+		Convergent: Overall Spearman correlation (95% CI) *r* = 0.78 (0.74–0.82); USA *r* = 0.72 (0.64–0.77); Denmark *r* = 0.85 (0.81–0.89)	
Kirby 2020^43^	Hidradenitis Suppurativa Quality of Life (HiSQOL)	Hidradenitis suppurativa	United States, Denmark	405		+				+		HiSQOL demonstrated very strong correlations between the HiSQOL total score and DLQI score (0.90). Known‐groups validity of HiSQOL across established DLQI score groups	Known‐groups validity was evaluated as the differences in HiSQOL scores among known scoring bands for the DLQI using ANOVA
Kocaturk 2012^93^	Chronic Urticaria Quality of Life Questionnaire (CU‐Q2oL) Turkish	Urticaria	Turkey	140		+					+	Correlation between the total scores of CU‐Q2oL and DLQI was highly significant (*r* = 0.77, *p* < 0.001)	
Koszoru 2023^44^	EQ5D 3L and 5L	Atopic dermatitis	Hungary	218		+				+		Convergent: EQ‐5D‐3L *r*s = 0.267 to 0.570 by EQ5D item; EQ‐5D‐5L *r*s = 0.354 to 0.670 by EQ5D item; EQ‐5D‐3L index *r*s = −0.669; EQ‐5D‐5L *r*s = −0.731	
Koszoru 2022^94^	Skindex‐16, EQ5D‐5L	Atopic dermatitis	Hungary	218		+				+		Convergent Skindex‐16 Total *r* = 0.827; Symptoms subscale *r* = 0.730; Emotions subscale *r* = 0.697; Functioning subscale *r* = 0.827; all *p* < 0.05. EQ5D‐5L *r* = −0.753 *p* < 0.05. Correlations between DLQI and Skindex and EQ‐5D. Relative efficiency determined as ratio of ESs of 2 HRQoL instruments, with DLQI as reference	Hypotheses confirmed regarding convergent validity. The EQ‐5D‐5L utilities had strong correlations with Skindex‐16 total and DLQI
Koti 2013^95^	Chronic Urticaria Quality of Life Questionnaire (CU‐Q2oL) Greek	Urticaria	Greece	110		+						Strong correlation between the total scores of the CU‐Q2oL and the DLQI (*r* = 0.75, *p* < 0.0001) and also between different scales of the Greek version of the CU‐Q2oL and DLQI subheadings: Functioning scale of the Greek CU‐Q2oL correlated with all DLQI subheadings, but showed the strongest correlation with DLQI subheadings: Leisure (*r* = 0.73, *p* < 0.0001), Daily activities (*r* = 0.57, *p* < 0.0001), Personal relationships (*r* = 0.53, *p* < 0.0001)	
Kottner 2013^96^	Person‐Centred Dermatology Self‐care Index (PeDeSI)	‘Any skin disease’	Germany	100		+					+	Correlation between PeDeSI‐G and DLQI sum scores was *r* = −0.287	
Krishna 2013^97^	Vitiligo Impact Scale (VIS)	Vitiligo	India	100		+				+		*r* = 0.65	
Kulthanan 2019^45^	Angioedema quality of life questionnaire (AE‐QoL)	Urticaria	Thailand	86		+				+		The AE‐QoL showed high convergent validity, with strong positive correlations between AE‐QoL and DLQI total score values (*r* = 0.72, *p* < 0.0001). Known group: The AE‐QoL also showed high known‐groups validity as it was able to discriminate among patients who showed differences in HRQoL impairment as assessed by the use of the PGA‐QoL and the DLQI	Known group: patients were classified into three groups using the DLQI and PGA‐QoL scores in this study, as follows: (i) ‘no effect’ (DLQI scores of 0–1, PGA‐QoL score of 0); (ii) ‘small effect’ (DLQI scores of 2–5, PGA‐QoL score of 1); and, (iii) ‘moderate to large effect’ (DLQI scores of 6–30, PGA‐QoL scores of 2–4)
Kulthanan 2016^46^	Chronic Urticaria Quality of Life Questionnaire (CU‐Q2oL) Thai	Urticaria	Thailand	166	+						+	Construct: Strong correlation between total DLQI score and total CU‐Q2oL score (*r* = 0.76, *p* < 0.0001). Correlations between each corresponding domain of DLQI and CU‐Q2oL were strong and statistically significant (all *r* values ≥ 0.63, *p* < 0.0001). Known group: statistically significant differences in the CU‐Q2oL total score among five DLQI groups were found (*p* < 0.0001)	Known group (Kruskal–Wallis test): DLQI scores were used to differentiate five levels of HRQoL impairment based on Hongbo bands: no effect (score of 0–1), small effect (score of 2–5), moderate effect (score of 6–10), large effect (score of 11–20) and extremely large effect (score of 21–30)
Larsen 2021^98^	Hidradenitis Suppurativa Quality of Life (HiSQOL)	Hidradenitis suppurativa	Denmark	103		+				+		Very strong and statistically significant correlation was found between HiSQOL and DLQI (*q* = 0.93, *p* < 0.0001, (95% CI: 0.89; 0.95)). Sub‐analysis (score > 2 in domain 10–12 in HiSQOL, *n* = 35) showed lower correlation coefficients; however, the strength of the correlations remained the same and remained statistically significant: HiSQOL and DLQI (*q* = 0.89, *p* < 0.0001 (95% CI: 0.78; 0.94))	
Law 2009^99^	Cardiff Acne Disability Index (CADI) Chinese	Acne	China	0					+	+		Criterion validity: Chinese CADI and DLQI were strong (γs = 0.58) and significant (*p* = 0.004). The strength of the relationship between the Chinese CADI and Cantonese DLQI was also large (γs = 0.72) and significant (*p* < 0.001)	
Lilly 2013^100^	VitiQol (Vitiligo‐specific health‐related quality of life instrument)	Vitiligo	United States	90		+					+	Convergent validity was demonstrated by large, significant (*p* < 0.01) correlations between VitiQoL and DLQI, *r* = 0.832	
Liyanage 2022^101^	Psoriasis Disability Index (PDI) Sinhala	Psoriasis	Sri Lanka	199		+				+		PDI and DLQI scores showed good correlation *r* = 0.76 (*p* < 0.01)	
Lockhart 2013^102^	Vulval Intraepithelial Neoplasia (VIN) questionnaire	Vulval intraepithelial neoplasia	United Kingdom	58		+				+		The VIN questionnaire score was significantly correlated with the DLQI (*r* = 0.69)	
Maranzatto 2016^103^	Melasma quality of life scale (MELASQoL‐Brazilian Portuguese)	Melasma	Brazil	154		+				+		Strong correlation (rho) between MELASQoL‐BP and DLQI: 0.70 (*p* < 0.01)	
Marron 2021^104^	Hidradenitis Suppurativa Quality of Life‐24 (HSQoL‐24)	Hidradenitis suppurativa	Spain	130	+							Correlation and regression analysis. Correlation with DLQI 0.690 (*p* < 0.001)	
McKenna 2003^105^	Psoriasis Index of Quality of Life (PSORIQoL)	Psoriasis	United Kingdom, Italy, Netherlands	148		+				+		Convergent: DLQI correlation with PSORIQoL 0.70; DLQI subscales: Symptoms and feelings 0.55 Daily activities 0.66 Leisure 0.53 Work and school 032, Personal relationships 0.45, Treatment 0.47	
Meier 2018^106^	Actinic Keratosis Quality of Life questionnaire	Actinic keratosis	Switzerland	106		+				+		The Spearman correlation coefficient between the total score of the AKQoL (*n* = 108) and the DLQI (*n* = 106) for our validation group was 0.57 with a significance level of *p* < 0.001	
Meneguin 2021^107^	Skindex‐16	Any skin disease	Brazil	188		+				+		High correlations can be seen between total Skindex‐16 score and DLQI‐BRA (0.75). Skindex‐16 domains: Sk‐16 s *r* = 0.57, Sk‐16 e *r* = 0.66, Sk‐16 f r = 0.70	
Mikoshiba 2015^108^	Hand–foot syndrome (HFS‐14)	Hand/foot syndrome	Japan	0			+			+		Spearman's rank correlation coefficient between score on the Japanese version of the HFS‐14 and score of 0.61 (*p* < 0.0001), respectively	
Milutinovic 2017^109^	Skindex‐29 Serbian	Acne vulgaris, 48 verrucae vulgaris, 40 psoriasis, 34 undetermined type of mild dermatitis, 20 venous ulcers, 19 eczema and 71 other skin diseases (acne rosacea, urticaria, keratosis, tinea corporis or pedis, scabies and others)	Serbia	586					+	+		Construct validity (Mann–Whitney) Criterion validity (Spearman's): emotional domain of Skindex‐29 *r* = 0.581, Symptoms domain of Skindex‐29 *r* = 0.501, Functioning domain of Skindex‐29 *r* = 0.554 (both first rating); all *p* < 0.001	
Minamoto 2018^110^	The Quality of Life in Hand Eczema Questionnaire (QOLHEQ) Japanese	Eczema/Hand eczema	Japan	124		+				+		Total QOLHEQ score showed strong correlation with DLQI (*r* = 0.71, *p* < 0.001)	
Mlynek 2009^111^	Chronic Urticaria Quality of Life Questionnaire (CU‐Q2oL) German	Urticaria	Germany	157		+					+	All of these two‐tailed correlations were highly significant at *p* < 0.001, confirming that five of the six German scales of the CU‐Q2oL have strong convergent validity with other established QoL questionnaires	
Morice‐Picard 2018^112^	Burden of albinism (BoA)	Albinism		63			+				+	The BoA questionnaire was highly correlated with the SF‐12, RSES and DLQI‐validated questionnaires	
Muhleisen 2009^28^	Pictorial representation of illness and self‐measure (PRISM)	Dermatitis (*n* = 71), Psoriasis (*n* = 36), Leg ulcer (*n* = 28), Tumour (*n* = 26)	Switzerland	227		+				+		Overall PRISM scores correlated well with DLQI *n* = 235 (*r* = −0.304; *p* < 0.001) Spearman's rank correlations for psoriasis *n* = 36 (ρ = −0.418, *p* < 0.01) and tumour *n* = 26 (ρ = −0.399, *p* < 0.04), dermatitis *n*‐71 (ρ = −0.371, *p* < 0.001), leg ulcer *n*‐28 (ρ = −0.288, *p* < 0.14)	
Muller 2017^113^	EORTC Core QL Questionnaire – Cancer (QLQ‐C30)	Non‐melanoma skin cancer	Germany	169	+	+				+		DLQI total score was significantly associated with all functioning and symptom scales of the QLQ‐C30, ranging from *r* (s) = 0.16 to 0.49. Substantial correlations (*r* (s) ≥ 0.40) were found between the DLQI total score and QLQ‐C30 subscales role, emotional and social functioning as well as with global quality of life	
Nochaiwong 2017^114^	Uraemic Pruritus in Dialysis Patients (UP‐Dial)	Uraemic pruritus	Thailand	168		+		+		+		Convergent validity Correlation with DLQI (95% CI): Summary score 0.78 (0.71–0.85); Signs and symptoms 0.63 (0.53–0.73); Psychosocial 0.80 (0.72–0.87); Sleep 0.53 (0.43–0.64). Discriminant validity: UP‐Dial Significantly less than the DLQI: 21% (three of 14 items) vs. 70% (seven of 10 items); *p* = 0.035	
Nochaiwong 2018^115^	Uraemic Pruritus in Dialysis Patients (UP‐Dial)	Uraemic pruritus	Thailand	258		+				+		The UP‐Dial scale correlation with DLQI: *r* = 0.79. Subscale (95% CI): signs and symptoms 0.70 (0.63–0.76) Subscale: psychosocial 0.76 (0.69–0.83) Subscale: sleep. Dermatology‐specific QOL: DLQI 0.70 (0.63–0.76) 0.76 (0.69–0.83) 0.57 (0.49–0.65) 0.79 (0.73–0.84) Summary score Dermatology‐specific QOL: DLQI 0.70 (0.63–0.76) 0.76 (0.69–0.83) 0.57 (0.49–0.65) 0.79 (0.73–0.84)	The UP‐Dial scale correlation with anchor‐based questions
Ofenloch 2015^116^	Occupational Contact Dermatitis Disease Severity Index (ODDI)	Contact dermatitis	Germany	422	+					+		Correlations between ODDI total and DLQI Index (95% CI): Overall *r* = 0.36 (0.28–0.44); ODDI disease severity 0.35 (0.26–0.43); ODDI work limitations 0.31 (0.22–0.40)	
Oosterhaven 2020^29^	Quality of Life in Hand Eczema Questionnaire (QOLHEQ) Dutch	Eczema/Hand eczema	Netherlands	300		+					+	Single‐score validity (at T0) correlations between the Quality of Life in Hand Eczema Questionnaire (QOLHEQ) correlation found 0.77, *R*2 = 0.59	
Paula 2014^117^	Skindex‐29	Any skin disease	Brazil	75		+				+		Total Skindex scores *r* = 0.780, Emotions *r* = 0.665, Symptoms *r* = 0.622, Functioning *r* = 0.803, *N* = 75 all *p* < 0.01	
Peris 2019^118^	HIDRAdisk	Hidradenitis suppurativa	Italy	140	+					+		HIDRAdisk showed a strong correlation with DLQI *r* = 0.6651, *p* < 0.0001	
Polking 2018^119^	Prurigo Activity Score (PAS)	Prurigo nodularis	Germany	12		+	+			+		Convergent (Spearman's): PAS items 1b (predominant lesion type), 2 (estimated number) and 7a (excoriations/crusts) showed good correlations with mostly high significances to DLQI	Concurrent: Kruskal–Wallis for difference between Hongbo bandings The DLQI achieved the best predictive and adaptive quality compared with the pruritus intensity scales (NRS and VAS)
Pollo 2018^120^	Melasma quality of life scale (MELASQoL‐Brazilian Portuguese), HRQ‐Melasma	Melasma	Brazil	154			+			+		There was high correlation between HRQ‐Melasma and DLQI and MELASQoL‐BP (ρ = 0.80 and 0.83)	
Poór 2017^121^	EQ5D 3L and 5L	Psoriasis	Hungary	238		+				+		Correlation analysis between DLQI and the two EQ‐5D versions revealed better convergence of 5L compared with the 3L for mobility (0.224 vs. 0.226), self‐care (0.342 vs. 0.368) and usual activities (0.479 vs. 0.496) dimension. The 3L indicated a slightly better convergent validity with EQ VAS with the exception of the usual activities (−0.462 vs. −0.512) dimension. Moderate correlations were observed between the dermatological measures (PASI, DLQI) and both EQ‐5D index scores, as well as with the EQ VAS	
Puelles 2022^30^	9SD‐NRS (Sleep Disturbance Numerical Rating Scale)	Atopic dermatitis	United States, Australia, Canada, Germany, Poland, France	207		+				+		Convergent: *N* = 207 *r* = 0.42 *p* < 0.001 for DLQI	Known‐groups validity: comparison of mean SD NRS average weekly scores at baseline by DLQI total score using ANOVA adjusted for multiple comparisons based on the Scheffe method
Reich 2017^122^	12‐Item Pruritus Severity Scale (12‐PSS)	Pruritus	Poland	148		+					+	Convergent: *r* = 0.53, *p* < 0.001. Known group (ANOVA): 12‐PSS was able to detect significant differences between patients having various levels of QoL impairment assessed according to DLQI (*p* < 0.001)	
Rencz 2022^47^	ICECAP‐A	Multiple skin diseases	Hungary	618		+				+		DLQI was weakly correlated with all five ICECAP‐A attributes and index score (*r*s = − 0.271); Stability −0.236, Attachment −0.200, Autonomy −0.201, Achievement −0.182, Enjoyment −0.220. Known group: Mean ICECAPA index scores of patients with a DLQI ≤10 and DLQI >10 were 0.70 ± 0.19 and 0.61 ± 0.23, respectively (*p* = 0.002)	
Rentz 2020^48^	Psoriasis Symptom Scale (PSS)	Psoriasis	Australia, Austria, Belgium, Canada, Czech Republic, France, Germany, Japan, Mexico, Poland, Portugal, South Korea, Spain, United States	1000		+					+	At baseline, the PSS total score and DLQI were moderately to strongly correlated, correlations ranged from 0.33 to 0.66, *p* < 0.001. Higher correlations were seen at week 16 (0.57 to 0.80), except between the PSS and the work and school dimension (0.31), all *p* < 0.001	Evidence of known‐groups validity (ANCOVA) was shown with a significant association between the PSS total score and two‐category DLQI scores (0–1, ≥2) at weeks 12 and 16 (*p* < 0.0001)
Rhee 2006^123^	Skin Cancer Index	Non‐melanoma skin cancer	United States	228		+		+		+		Score Correlation of Standardized Skin Cancer Index: Total −0.340, Emotional −0.291, Social −0.349, Appearance −0.288; all *p* < 0.001	Convergent and divergent validity were assessed by computing a Spearman correlation coefficient between the standardized SCI items and each of SF‐12; Lerman's Cancer Worry Scale; DLQI); Rosenberg Self‐Esteem Scale; and Marlowe‐Crowne Social Desirability Scale
Salzes 2016^124^	Vitiligo Impact Scale	Vitiligo	Not specified	301			+			+		Correlation of total DLQI score with VIP: Dark skin phototypes: *r* = 0.84; Fair skin phototypes *r* = 0.82, both *p* < 0.05	Concurrent validity was determined by calculating the Spearman coefficient (*r*) and Bland and Altman plots between VIPs‐FS/VIPsDS and the four other distributed questionnaires
Sampogna 2015^49^	PSOdisk	Psoriasis	Italy	320		+					+	Convergent correlation *r* = 0.715. Known group (statistical method not stated): for the lowest and the highest categories the association was very strong, while for the middle categories, the distribution was more widespread	Known group: comparison between PSOdisk categories (<9, 9–15, 16–30, 31–50, >50) and DLQI Hongbo categories (0–1, 2–5, 6–10, 11–20, 21–30) at baseline
Saunderson, 2020^125^	Vulvar Quality of Life Index (VQLI)	Vulvar disease: Lichen sclerosis, Recurrent vulvovaginal candidiasis, Lichen planus, Vulvodynia, Lichen simplex chronicus	Australia	243		+				+		The total VQLI strongly correlated with the total DLQI score (0.89)	
Schwartzman 2021^31^	Patient‐Reported Outcomes Measurement Information System Global Health (PGH)	Atopic dermatitis	United States	994			+	+		+		PGH‐P4 T scores had moderate correlation with DLQI: PGH‐P4 T score −0.40, PGH‐M4 T score −0.36, PGH‐P2 T score −0.29, PGH‐M2 T score −0.30; all *p* < 0.001. mEQ‐5D, PGH‐P4, PGH‐M4 and PGH‐M2T scores and DLQI scores showed similar multilevel areas under the curve, indicating poor known‐groups validity in predicting self‐reported global atopic dermatitis severity overall	Known groups tested with were established with logistic regression models with self‐reported global atopic dermatitis severity as the ordinal dependent variable and AUC
Sebaratnam 2013^126^	Autoimmune Bullous Disease Quality of Life (ABQOL)	Bullous disease	Australia	70		+		+		+		Convergent validity: Correlation with DLQI *R* = 0.64. Discriminant validity: Comparison of sensitivity with DLQI (Fisher exact test) *p* < 0.02	
Şenol 2013^127^	VLQI (Vitiligo Life Quality Index)	Vitiligo	Turkey	178		+		+				VLQI was correlated (statistical test not given) with DLQI indicating convergent validity (*r* = 0.77, *p* < 0.001). Discriminant validity results not given	Discriminant validity: Mann–Whitney *U*‐test or Kruskal–Wallis analysis
Shimizu 2018^128^	WAA‐QoL (Women's Androgenetic Alopecia Quality of Life Questionnaire)	Alopecia	Brazil	116		+				+		The correlation between WAA‐QoL and DLQI resulted in (rho) 0.81 (*p* < 0.01)	
Shourick 2022^129^	Vitiligo Treatment Impact score (VITs)	Vitiligo	France	343	+							The 19‐item questionnaire correlated moderately with the DLQI 0.593 [CI 0.511, 0.665]	
Sibaud 2011^130^	Hand–foot syndrome (HFS‐14)	Hand/foot syndrome	France	39		+				+		HFS‐14 score was positively correlated with the DLQI scores, with highly significant consistency (*r* = 0.713; *p* < 0.0001)	
Silverberg 2019^131^	Short Form (SF)‐12	Atopic dermatitis	United States	602		+		+			+	Correlation of DLQI with SF‐12 MCS *r* = −0.441, SF‐12 PCS *r* = −0.06, both *p* < 0.0001. The DLQI had better convergent and discriminant validity than the SF‐12, particularly at distinguishing between moderate versus mild and severe versus moderate AD	Discriminant construct validity was determined using Wilcoxon rank sum tests. The DLQI had stronger correlations with the POEM, POSCORAD, PO‐SCORAD and the Numerical Rating Scale for pain than with the SF‐12 MCS, SF‐12 PCS or SF‐6D (*z*‐test, *p* < 0.001 for all). Regarding discriminative validity, there were significant decreases in the SF‐12 MCS and the SF‐6D scores, and increases in DLQI scores
Silverberg 2020^32^	PROMIS Itch Questionnaire Mood and Sleep (PIQ‐MS)	Atopic dermatitis	United States	410		+				+		Convergent: Baseline DLQI correlations: NRS worse 0.51, NRS average 0.53, VRS worse 0.46. NRS average 0.48. frequency of itch 0.53, all *p* < 0.001	
Silverberg 2021^33^	Patient Health Questionnaire‐9 (PHQ‐9), Abridged version Patient Health Questionnaire (PHQ‐2)	Atopic dermatitis	United States	458		+				+		PHQ‐9 was strongly correlated with DLQI (*r* = 0.50) and PHQ‐2 (*r* = 0.48), *p* < 0.001 for all, *N* = 548	
Simpson 2019^34^	Atopic Dermatitis Control Tool (ADCT)	Atopic dermatitis	United States	2584		+				+		Convergent: DLQI total score correlations: Baseline 0.543, month 1 0.803, month 2 0.828, month 3, 0.846, month 6, 0.812, all *p* < 0.001. Almost all differences in mean ADCT total scores between the adjacent bands were statistically significant (*p* < 0.05). Known group: patients in the groups of DLQI bands with greater effect on life were associated with higher mean ADCT total scores (poor AD control). Almost all Cohen's d effect size showed large effect across all adjacent categories	Known group (Mann–Whitney *U*‐test or *t*‐test): mean ADCT total scores (baseline, months 1, 2, 3, 6) were compared across adjacent subgroups of patients based on categories of DLQI responses: no effect on patient life (score range: 0–1)
Stepien 2020^132^	12‐Item Pruritus Severity Score (12‐PSS)	Pruritus	Poland	202		+				+		DLQI total scores demonstrated good correlation with 12‐PSS scores (ρ = 0.54)	
Strober 2016^35^	Psoriasis Symptom Diary	Psoriasis	Multiple worldwide	820		+						Construct validity correlations at week 12 (*n* = 722–724): PSD item vs DLQI total; 1. Itching 0.71, 3. Stinging 0.70, 5. Burning 0.69, 7. Pain/cracking 0.70, 9. Pain 0.69, 11. Scaling 0.72, 13. Notice – colour 0.67; all correlations significant *p* < 0.01	Correlation method not given
Taieb 2015^133^	Atopic Dermatitis Burden Scale for Adults (ABS‐A)	Atopic dermatitis	France	128			+			+		The overall ABS‐A score showed very good correlation with the DLQI score (*r* = −0.78). Daily life 0.73, *p* < 0.0001; Economic constraints 0.58, *p* < 0.0001; Care and management of disease 0.33, *p* = 0.0004; Work and stress 0.66, *p* < 0.0001	
Tamasi 2019^134^	EQ‐5D	Pemphigus	Hungary	109		+						Correlations −0.62 with Equation 5D and −0.42 with EQ VAS	EQ‐5D‐5L index scores demonstrated a strong correlation with DLQI. EQ VAS moderately correlated with the DLQI and the scores on pain intensity scales (*p* < 0.0001)
Tawil 2020^135^	Chronic Urticaria Quality of Life Questionnaire (CU‐Q2oL, Arabic version), Arabic version of UAS 7	Urticaria	Lebanon	152		+					+	CU‐Q2oL total score strongly correlated with the DLQI score (*r* = 0.86, *p* < 0.001). Domain I 12, 11, 7, 21, 13, 20, 5, 19, 22 *r* = 0.81*; Domain II 10, 17, 23, 9, 8, 16 *r* = 0.75*; Domain III 14, 18, 15, 6 *r* = 0.71*; Domain IV 3, 4 *r* = 0.51*; Domain V 1, 2 0.58*; *p < 0.001	
Temel 2019^136^	Internalized Stigma Scale (ISS)	Acne, Alopecia Areata, Vitiligo	Turkey	150		+						Significant correlation between ISS and DLQI (*r* = 0.596, *p* < 0.001) for patients with acne, significant correlation between ISS and DLQI (*r* = 0.540, *p* < 0.001) in patients with vitiligo. Significant correlation between ISS and DLQI (*r* = 0.508, *p* < 0.001) for patient with alopecia areata	
Tjokrowidjaja 2013^137^	Autoimmune Bullous Disease Quality of Life (ABQOL)	Bullous disease	Australia	70		+						Moderate correlation with DLQI (*r* = 0.64)	
Tosun 2022^138^	Multidimensional Assessment of Interoceptive (bodily sensations) Awareness‐2 (MAIA‐2)	124 acne vulgaris, 57 psoriasis vulgaris, 50 dermatitis, 32 telogen effluvium, 22 pruritus, 20 urticaria, 12 tineas, 9 lichen planus, 8 nevus, 7 rosacea, 7 verruca and 42 patients diagnosed with other diseases	Turkey	390	+	+						Differences between MAIA‐2 and DLQI scale levels according to diagnosis were analysed by ANOVA; MAIA‐2 and DLQI Scales exhibited significant differences according to the diagnosis (*p* < 0.05) for psoriasis, pruritis, tinea, verruca, urticaria and other diseases	MAIA‐2 Scale affects the DLQI Scale (β = 0.853; *p* < 0.05) positively, F1 (Attention Regulation) subscale affects it negatively (β = −0.324; *p* < 0.05), F5 (Self‐regulation) subscale affects it negatively (β = −0.328; *p* < 0.05) and F6 (Trusting) subscale affects it negatively (β = −0.206; *p* < 0.05). Chi^2^/df = 2.252, RMSEA = 0.058, NFI = 0.807, NNFT(TLI) = 0.874, CFI = 0.882
Vitala 2008^139^	CECA10 (Specific Questionnaire for Condylomata Acuminata) QOL	Condylomata acuminata (warts)	Spain	247		+				+		Correlation between CECA and DLQI scores ranged from moderate to high. The emotional and personal relationships dimensions of the DLQI showed the highest correlations with the emotional dimension (*r* ^2^ = −0.528) and the sexual activity dimension (*r* ^2^ = −0.673) of the CECA as they measured the same HRQoL aspects	
Warren 2021^37^	Psoriasis Symptoms and Impacts Measure (P‐SIM)	Psoriasis	United States, Canada, Belgium, Germany, Italy, United Kingdom, Hungary, Poland, Russian Federation, Australia, Japan, Korea	1002	+	+				+		All P‐SIM items were moderately to strongly correlated with DLQI total score and DLQI item 1 score at baseline and week 16. As expected, P‐SIM items 1, 3, 4 and 8 (itching, skin pain, burning and irritation, respectively) were strongly correlated with DLQI item 1 score at both time points	
Whalley 2004^140^	Quality of Life Index for Atopic Dermatitis (QoLIAD)	Atopic dermatitis	United Kingdom, United States, Netherlands, France, Germany, Italy	1750		+				+		Correlations between the QoLIAD and DLQI ranged from 0.65 to 0.79 at time 1 and from 0.58 to 0.77 at time 2	
Wulandani 2018^141^	5‐D Itch scale	Pruritus	Indonesia	0		+						Convergent: Disability domain of 5DIS and DLQI *r* = 0.563 *p* = 0.001	
Xavier 2022^142^	Skin picking disorder (SPD, Brazilian)	Skin picking disorder (SPD)	Brazil	124			+				+	Concurrent validity demonstrated correlation with the DLQI (*r* = 0.73)	
Yeung 2015^143^	Cutaneous Sarcoidosis Activity and Morphology Instrument (CSAMI), Sarcoidosis Activity and Severity Index (SASI)	Sarcoidosis	United States	13		+				+	+	CSAMI Activity scale demonstrated a strong Spearman's correlation with the DLQI (ρ = 0.70 [95% CI, 0.25–0.90]) and moderate Pearson's correlation with the SAT Skin Stigma raw score (*r* = 0.56 [95% CI, 0.01–0.85])	
Yfantopoulos 2017^50^	EQ5D 3L and 5L	Psoriasis	Greece	396		+				+		Correlations between EQ‐5D dimensions and DLQI sum score were all significant at least at alpha = 5%. EQ‐5D‐5L items were stronger correlated with the DLQI sum score (mean q5L = 0.210 vs. q3L = 0.192, *p* = 0.039 based on a paired *t*‐test), with the largest discrepancy found in ‘usual activities’ (*p* = 0.060 for the difference in coefficients). Overall, the EQ‐5D‐5L items demonstrated marginally better convergent validity (mean q5L = 0.138 vs. q3L = 0.122, *p* < 0.001 based on a paired *t*‐test	ROC: DLQI No effect (*N* = 65) vs. Small, moderate, very large and extremely large effect (*N* = 331) *p* = 0.984; No effect and small effect (*N* = 225) vs. Moderate, very large and extremely large effect (*N* = 171) *p* = 0.448; No effect, small and moderate effect (*N* = 309) vs. Very large and extremely large effect (*N* = 87) *p* = 0.121; No effect, small, moderate and very large (*N* = 385) vs. Extremely large effect (*N* = 11) *p* = 0.166. ROC curves reaffirmed the known‐groups validity for both EQ‐5D instruments with respect to health state, severity of disease and impact of psoriasis. Both instruments were able to differentiate effectively between groups known to differ clinically
Zeidler 2019^144^	ItchyQoL	Any skin disease	Austria, France, Germany, Italy, Poland, Russia, Spain, Switzerland, Turkey	535		+	+			+		DLQI score strongly correlated with ItchyQoL (all *r* = 0.72. *p* = 0.001). Subscores ‘functioning’ (*r* = 0.71, *p* < 0.001) and ‘emotions’ (*r* = 0.69, *p* < 0.001). A moderate correlation between DLQI score and subscore ‘symptoms’ (*r* = 0.47, *p* < 0.001) was observed. Concurrent: There were statistically significant differences in patients with ‘no effect’ (DLQI: 0–1), ‘small’ (DLQI 2–5), ‘moderate’ (DLQI 6–10), ‘very large’ (DLQI 11–19) and ‘extremely large effect on patient's life’ (DLQI 21–30)	
Zhao 2021^145^	Vitiligo‐specific quality of life instrument (VitiQoL) Chinese	Vitiligo	China	182		+		+			+	Discriminant validity *p* = 0.076 (there was no significant difference in the proportion of insensitive items between VitiQoL and DLQI). Convergent validity *r* = 0.70 (*p* < 0.01)	Discriminant validity: Pearson's Chi‐square test
Zhao 2022^146^	Chronic urticaria quality of life questionnaire (CU‐Q2oL) Chinese	Urticaria	China	325		+				+		DLQI correlated with total CU‐Q2oL score (*r* = 0.769, *p* < 0.001). The CU‐Q2oL scale I (function/mental status) was correlated with the mean score of DLQI items 3, 5, 6 and 7/7a (*r* = 0.73, *p* < 0.001); the CU‐Q2oL scale III (Itching/bothered) was correlated with the mean score of DLQI items 1 and 2 (*r* = 0.507, *p* < 0.001). The CU‐Q2oL scale IV (Limits) was correlated with DLQI item 4 (*r* = 0.563, *p* < 0.001)	
Ortonne 2010^147^	Nail psoriasis quality of life scale (NPQ10)	Nail psoriasis	France	795	+							Correlation between NPQ10 and dimensions DLQI: Symptoms, feelings 0.38, Daily activities 0.46, Leisure 0.37, Work 0.44, Personal relationships 0.34, Treatment 0.24, DLQI total 0.48	

Seventy‐five (61.5%) of the 122 studies included in this review were validations of the original language versions of the PRO instruments (including 43 studies describing new instruments), with the rest (47, 38.5%) being cross‐cultural adaptations. The instruments with the highest number of adaptations using the DLQI for validation were the Chronic Urticaria Quality of Life Questionnaire (CU‐Q2oL, 9 studies), the Cardiff Acne Disability Index (CADI, 3 studies) and Skindex (2 studies). Seven studies (5.7%) were of comparisons among existing translated instruments.

Some of the 122 studies included in this review should be considered validations but used alternative methods to those already presented. For example, the study of Pickard et al.^148^ used EQ‐5D health utilities to explore ways to improve responsiveness in psoriasis. It determined that EQ‐5D health utility scores were able to discriminate among different levels of improvement in psoriasis severity following therapy, using stratification by baseline DLQI > 10.

Additionally, Yildirim et al.^149^ conducted a Cross‐European validation of the ItchyQoL in pruritic dermatoses to assess the impairment of health‐related quality of life (HRQoL) in patients with psoriasis. They reported the validation in several languages across Europe using the DLQI and a multiple linear regression model to determine factors which were most relevant on the Liebowitz Social Anxiety Scale and the Hospital Anxiety and Depression Scale (HADS).

To determine the interpretability of the Thai CU‐Q2oL (the ability of an instrument to be interpreted from quantitative scores or changes in scores, with a qualitative meaning), the study of Kulthanan et al.^46^ used ROC and area under the curve (AUC) to investigate the ability of the Thai CU‐Q2oL to detect changes in patients' HRQoL impairment over time. The ROC analysis showed that the reduction in the Thai CUQ2oL score of ≥15 was the best defined MCID as both sensitivity (83.3%) and specificity (82.4%) of this value were high. Yfantopoulos et al.^50^ also used the area under ROC curves (AUC) to determine the discriminatory properties of the EQ‐5D‐3L and EQ‐5D‐5L instruments in psoriasis by external indicators of health (DLQI, PGA and VAS). Their findings of the ROC curves reaffirmed the known‐groups validity for both EQ‐5D instruments with respect to health state, severity of disease and impact of psoriasis; however, the EQ‐5D‐5L was more efficient in most comparisons.

Warren et al. used empirical cumulative distribution function (eCDF) curves of observed changes from baseline to week 16 in P‐SIM item scores by DLQI item 1 change‐score category. These supported a 4‐point responder division to represent marked clinical improvement and to define responders in patients with moderate‐to‐severe plaque psoriasis in clinical trials.

### Appraisal of representation of minoritized ethnic participants

The results of the analysis of patients included in studies by Naicker's Critically Appraisal Tool are shown in Table 6.

**TABLE 6:** Data from Naicker's Critically Appraising for Antiracism Tool.

Question	Yes	No	Unclear	N/A	Total
Were minoritized ethnic participants recruited?	17 (13.9%)	3 (2.5%)	102 (83.6%)	0	122
Were minoritized ethnic participants representative?	5 (4.1%)	2 (1.6%)	115 (94.3%)	0	122
Are the methodologies suitable/validated for minoritized ethnic population?	2 (1.6%)	0	1 (0.8%)	119 (97.5%)	122
Were results data stratified by race/ethnicity and if so, was this justified/appropriate/explained by the author?	1 (0.8%)	10 (8.2%)	0	111 (91.0%)	122
Were any differences in study outcomes for minoritized ethnic populations appropriately addressed and interpreted?	0	2 (1.6%)	0	120 (98.4%)	122
Did researchers avoid assigning race as a variable, a risk factor or a proxy for genetic ancestry?	0	2 (1.6%)	0	120 (98.4%)	122

*Note*: Naicker.^19^ Critically Appraising for Antiracism Tool. Available at: https://www.criticallyappraisingantiracism.org/.

## DISCUSSION

In the 1980s it was being recognized that the detrimental effect of skin diseases on the QoL of patients needed to be measured^150^, ^151^ with important implications for optimal patient management.^152^ Although disease‐specific indices of disability had been developed to record the impact of atopic eczema, psoriasis and acne,^153^, ^154^, ^155^ there was a need for a general dermatology‐specific QoL measure and a simple, compact, practical questionnaire technique for routine clinical use with a simple scoring process and easily interpretable results. The DLQI^156^ was developed, based on questionnaire development practice at the time, based on patients reporting of their most commonly experienced aspects of QoL impairment. The measure showed good test–retest reliability, good construct validity and a good degree of consistency of responses between questions. The DLQI is the PRO tool most widely used by clinicians and researchers to understand the burden of skin diseases on patients and to assess the effectiveness of interventions.^157^

Since the development of the DLQI, traditional classical test theory (CTT) has been substantially developed with a focus on validity and reliability (beyond simple measures such as Cronbach's alpha) and Generalizability Theory (G‐Theory),^158^ and there has been a significant shift towards the use of Differential Item Functioning (DIF)^159^ and Item Response Theory (IRT).^160^ All of these have been accompanied by much improved metrics, for example the reporting of the goodness‐of fit‐statistics.^161^ Most of these techniques have been applied to more recent validation studies of the DLQI as described in a previous systematic review that identified validation aspects of the DLQI^162^ described across 207 studies.

The concept of QoL^163^ has also greatly expanded since the development of the DLQI. Greater emphasis has been placed on subjective well‐being, recognizing that individuals' perceptions of their own lives are crucial.^164^ Measures of life satisfaction, happiness and personal fulfilment have become central to QoL assessments, alongside objective indicators. The role of sociocultural (cultural, social and community) factors are also increasingly recognized to play a role in QoL.^165^ Many QoL measures now take a holistic approach, shifting from purely economic or health‐based perspectives. However, the DLQI does not suffer from lack of these concepts, as they are of much less relevance to both patient and physician in the context of the healthcare consultation and treatment and management of specific dermatological diseases. Because the initial design of the DLQI captured those concepts that were important to patients, it has remained both relevant and comprehensive. Where needed, other measures are available that address these broader issues.

This systematic review compiles data from 122 peer‐reviewed studies describing research on 30,727 patients across 34 different countries with 41 diseases using the DLQI in the validation of 101 PRO‐QoL instruments, including 80 different dermatology‐specific QoL measures (mostly disease‐specific), and of 21 generic measures. Forty‐six (37.7%) studies were multicentre with 25.4% conducted at two or more sites. As expected, most studies (95.1%) did not involve any intervention as this complicates the analysis of validation.

More than a third of the studies (47, 38.5%) were cross‐cultural adaptations. This relates to the increasing awareness worldwide of the benefits of using PRO tools to enhance patient care, and the need for specific instruments in local languages. This is reflected by the 33 non‐English speaking countries where these studies were conducted. Although only 18 studies reported the specific language version of the DLQI that they used, it is highly likely that non‐English speaking countries used a local language version of the DLQI. Many studies (43) described the development of new instruments, using the DLQI in their validation process, a strong indication of the important role that the DLQI plays, particularly in the development of dermatologically focused instruments.

The proportion of studies that focussed on psoriasis as the study disease was lower than expected (*n* = 22, 15.3%), in view of the report of 55.3% psoriasis studies identified in a systematic review of 454 randomized controlled trials using the DLQI^157^ and 25.5% in a systematic review of 207 studies describing validation aspects of the DLQI.^162^ However, the range of diseases, including psoriasis, atopic dermatitis (11.1%), vitiligo (7.6%), urticaria (7.6%), hidradenitis suppurativa (5.6%) and acne (3.5%) partially mirrors the prevalence and burden, and therefore importance, of these diseases (percentage of global burden of disease measured in disability‐adjusted life years: dermatitis 0.38, acne 0.29, psoriasis 0.19 and urticaria 0.19).^166^ Of the publications studying psoriasis, 56% were validating a psoriasis specific instrument.

Known‐groups validity^38^ was tested in 14 studies: Such evidence is essential to provide confidence in the construct and use of a measure. Seven of these^27^, ^30^, ^43^, ^44^, ^45^, ^46^, ^49^ used the well‐established DLQI score descriptor bands of Hongbo et al.^39^ Know‐group analysis is an essential component of construct validity assessment as it provides evidence that the instrument is measuring what it intends to measure. Use of the DLQI as an anchor, particularly using Hongbo banding, indicates that researchers have confidence in the utility of the DLQI, even though a clinical severity scale, for example PASI, IGA and PGA might be easier to collect and use for this purpose but with significant limitations, particularly their linearity or lack of ability to discriminate small changes in severity.^167^

Correlation analysis allows the measure of the strength and direction of the relationships between scores of an instrument and other variables to assess various aspects of validity including construct, convergent, divergent, concurrent and criterion validity. Use of the DLQI in correlation analysis in 106 studies with many different PRO/QoL measures demonstrated the DLQI's utility in assessing construct (10), convergent (101), concurrent (10), divergent (10) and criterion validity (three studies). The majority of correlations were used in convergent validity to assess the degree to which the instrument correlated positively with the DLQI, chosen because its measures the same or similar constructs to the instruments being validated. This review has also demonstrated the use of the DLQI in validation over a wide variety of generic measures, including those measuring anxiety,^53^, ^149^ depression,^53^, ^149^ stigma,^27^, ^74^, ^78^, ^136^ acceptance of illness,^59^ fragrence allergy,^86^ and mood and sleep.

Like most measures developed in the 1990s (and most still today), the patient cohort used to develop the DLQI was not ethnically diverse. This is an issue that still needs addressing today in all measure development, and that we have highlighted in another publication^162^ using Naicker's Critical Appraisal Tool. Analysis using Naicker's Tool^19^ in this study revealed that only 13.9% of studies confirmed that minoritized ethnic participants were recruited; however, for the majority of studies (83.6%), recruitment policy was unclear. For the 17 studies that did recruit minoritized ethnic participants, only five appeared to show representative recruitment from the population. This may have been due to lack of reporting on the details of the recruited group (generally in the demographic data) or may have been due to very homogenous populations, for example in China and Japan, or the difficulty in determining race/ethnicity in very multiculturally complex countries, for example Brazil. However, even in those studies that did recruit minority races, differences in study outcomes for the minoritized ethnic populations were not addressed and interpreted, and researchers did not assign race/ethnicity as a variable, a risk factor or a proxy for genetic ancestry.

However, cross‐cultural adaptations also directly address this issue, and in the case of the DLQI there are for example, Canadian, Nigerian, Malaysian, Indian and USA cross‐cultural English language versions, 12 Arabic language versions, seven Chinese versions, seven French language versions, four German language versions, eight Russian versions and 10 Spanish language versions. Although some are only translations, most have been cross‐culturally adapted following accepted methods. In addition to cultural aspects considered in cross‐cultural adaptation of the DLQI, other aspects considered include ‘semantic’, and ‘experimental’ as well as conceptual.

The DLQI contains four items focusing mainly on the ability to feel (1, 2, 8 9) and six items mainly on the ability to do (3–7, 10), although certainly item 9 sexual difficulties (and perhaps others) could be characterized as both. As these items were generated from exhaustive patient perspectives of the issues important to them, the balance does not seem unusual and does not seem to be a limit in its design.

A large number of studies (188) found from searching the online databases were excluded because the participants were less than 16 years of age.^156^ This was because the DLQI was originally designed and validated for use with ages 16 years and older. Most of these studies utilized the Children's Dermatology Life Quality Index (CDLQI)^168^ and their inclusion resulted from our search strategy not including a method to sift these out. These rejected publications described the use of the CDLQI is the validation of several other childhood measures. In addition, a large number of studies (268) were excluded as they focused on disease severity scales, and not on validation of PRO/QoL measures. Disease severity/burden measures (objective parameters rated by clinicians) and PRO/QoL measures (assessed by patients) are different constructs and would not necessarily be expected to correlate: indeed, the reason that information from QoL measures may be helpful in informing clinical decisions is that in individuals QoL scores may be widely divergent from clinical objective scores. Disease severity is a clinical measure/outcome, not a patient‐reported measure/outcome, and thus was excluded from this study.

The data compiled and analysed by this study are not only of importance for researchers validating instruments, and for people searching for and assessing the use of measures for their research or routine clinical use, but they provide clinicians with greater assurance across the wide range of measures that we evaluated (disease‐specific or generic with different foci, e.g. depression, stigma, mood and sleep). These measures have some degree of connectivity, all being applied to dermatology patients, and all being validated against the same measure, the DLQI, which itself has achieved wide acceptance^157^ and broad validation.^162^ Thus, both clinicians and patients may be more confident about the appropriateness of their use in treatment decision‐making.

The strengths of this review include the large number of relevant articles identified, and the provision of direct access to previously scattered information. We are not aware of any similar exercise to bring together evidence of use of a QoL measure as a standard for comparison in validation of other dermatology instruments. A limitation of this study was to include only English language articles (19 articles found were excluded based on this criterion). In addition, some articles may not have been indexed in the databases we searched, as they prioritize English language journals and only index specific non‐English language journal articles if titles, keywords and abstracts are provided in English.

## CONCLUSIONS

This review identified widespread use of the DLQI as a benchmark in validation of other dermatology PRO/QoL measures and confirmed the central role DLQI plays in the development of novel instruments and validation across dermatology and beyond. The use of the DLQI by so many developers of other instruments has provided a common standard for validation. This makes it possible to compare aspects of validation across a wide range of QoL instruments. Developers of further novel PROMs for use in dermatology may thus benefit from including the DLQI as a comparator when planning their validation studies.

## AUTHOR CONTRIBUTIONS

AYF, JV, JRJ, FMA, JRI and SS made substantial contributions to conceptualization, design, validation and methodology. JV, JRJ, YA and FMA were responsible for acquisition and curation of data. AYF, SS and JRI were responsible for project administration and resources. AYF, SS, JRJ, JV and YA were responsible for formal analysis and interpretation of data. All authors were responsible for writing the original draft of the article or revising and reviewing it critically for important intellectual content. JRJ, JV, JRI, FMA, SS and AYF were responsible for the final approval of the version to be published.

## FUNDING INFORMATION

Funding was provided by the Division of Infection and Immunity, School of Medicine, Cardiff University, Cardiff, UK.

## CONFLICT OF INTEREST STATEMENT

Andrew Y Finlay is joint copyright owner of the Dermatology Life Quality Index (DLQI). Cardiff University receives royalties from some use of the DLQI: AYF receives a proportion of these under standard university policy. John Ingram receives a stipend as Editor‐in‐Chief of the British Journal of Dermatology and an authorship honorarium from UpToDate. He is a consultant for Abbvie, Boehringer Ingelheim, ChemoCentryx, MoonLake, Novartis, UCB Pharma and Union Therapeutics, and has served on advisory boards for Insmed, Kymera Therapeutics and Viela Bio. He is co‐copyright holder of HiSQOL, Investigator Global Assessment and Patient Global Assessment instruments for HS. His department receives income from royalties from the DLQI and related instruments. Sam Salek has received an unrestricted educational grant from GSK, is a consultant for Novo Nordisk and produces educational materials for Abbvie. Jui Vyas participated in an Advisory Board for Amgen, has received payment or honoraria from L'Oreal and support from UCB pharma for attending meetings. Faraz Ali has received honorariums from Abbvie, Janssen, LEO pharmaceuticals, Lilly pharmaceuticals, L'Oreal, Novartis and UCB. His department receives income from royalties from the DLQI and related instruments. Jeffrey Johns has no conflicts of interest to report. His department receives income from royalties from the DLQI and related instruments. Yasmina Abdelrazik has no conflicts of interest to report.

## ETHICAL APPROVAL

No ethics approval was required for this study.

## Supporting information


Table S1:

Appendix S1:

Appendix S2:

## Data Availability

All data are presented within this manuscript or the associated supplementary tables.
