# Supplementary material for: The Dermatology Life Quality Index (DLQI) used as the benchmark in validation of 101 quality‐of‐life instruments: A systematic review
Source: J Eur Acad Dermatol Venereol. 2024 Sep 13;39(3):631–79. doi: 10.1111/jdv.20321 (PMC11851266; doi:10.1111/jdv.20321)
Supplement: Supplementary file 1 — Table S1: [file JDV-39-631-s001.docx]

Supplementary Table 1 PRO-QoL Instruments and Diseases.

| **No. of studies/ refs** | **PRO- QoL instrument** |  | **Disease** |
| --- | --- | --- | --- |
| 2^122, 132^ | 12-Item Pruritus Severity Scale (12-PSS |  | Pruritus |
| 1^72^ | 12-item Psoriatic Arthritis Impact of Disease (PsAID-12)^b^ |  | Pruritus |
| 1^141^ | 5-D Itch scale |  | Pruritus |
| 1^74^ | 6-item Stigmatization |  | Generic |
| 1^30^ | 9SD-NRS (Sleep Disturbance Numerical Rating Scale) |  | Generic |
| 1^59^ | Acceptance of Illness Scale and Person-Centered Dermatology Self-care Index |  | General dermatological |
| 2^51, 106^ | Actinic Keratosis Quality of Life questionnaire |  | Acitinic keratosis |
| 1^45^ | Angioedema quality of life questionnaire (AE-QoL) |  | Urticaria |
| 1^133^ | Atopic Dermatitis Burden Scale for Adults (ABS-A) |  | Atopic dermatitis |
| 1^34^ | Atopic Dermatitis Control Tool (ADCT) |  | Atopic dermatitis |
| 1^53^ | Atopic Eczema Score of Emotional Consequences (AESEC) |  | Eczema/Hand eczema |
| 4^56, 67, 126, 137^ | Autoimmune Bullous Disease Quality of Life (ABQOL) |  | Bullous disease |
| 1^112^ | Burden of albinism (BoA) |  | Albinism |
| 1^99^ | Cardiff Acne Disability Index (CADI) Chinese |  | Acne |
| 2^83, 139^ | CECA10 (Specific Questionnaire for Condylomata Acuminata) QOL |  | Condylomat aacuminata |
| 1^135^ | Chronic Urticaria Quality of Life Questionnaire (CU-Q(2oL) Arabic |  | Urticaria |
| 1^73^ | Chronic Urticaria Quality of Life Questionnaire (CU-Q2oL) Brazilian-Portuguese |  | Urticaria |
| 1^79^ | Chronic Urticaria Quality of Life Questionnaire (CU-Q2oL) Portuguese |  | Urticaria |
| 1^146^ | Chronic urticaria Quality of Life questionnaire (CU-Q2oL) Chinese |  | Urticaria |
| 1^111^ | Chronic Urticaria Quality of Life Questionnaire (CU-Q2oL) German |  | Urticaria |
| 1^95^ | Chronic Urticaria Quality of Life Questionnaire (CU-Q2oL) Greek |  | Urticaria |
| 1^91^ | Chronic Urticaria Quality of Life Questionnaire (CU-Q2oL) Hebrew |  | Urticaria |
| 1^61^ | Chronic Urticaria Quality of Life Questionnaire (CU-Q2oL) Polish |  | Urticaria |
| 1^46^ | Chronic Urticaria Quality of Life Questionnaire (CU-Q2oL) Thai |  | Urticaria |
| 1^93^ | Chronic Urticaria Quality of Life Questionnaire (CU-Q2oL) Turkish |  | Urticaria |
| 1^143^ | Cutaneous Sarcoidosis Activity and Morphology Instrument (CSAMI) |  | Sarcoidosis |
| 1^64^ | Dermatomyositis Skin Severity Index (DSSI) |  | Dermatomyositis |
| 1^113^ | EORTC Core QL Questionnaire - Cancer (QLQ-C30) |  | Cancer |
| 4^44, 50, 57, 121^ | EQ5D 3L and 5L |  | Generic |
| 4^81, 94, 134, 148^ | EQ-5D-5L |  | Generic |
| 1^74^ | Feelings of Stigmatization Questionnaire |  | Generic |
| 1^86^ | Fragrance QoL instrument (FQL index) |  | Generic |
| 1^54^ | Freiburg Life Quality Assessment (FLQA) |  | Generic |
| 2^108, 130^ | Hand-foot syndrome (HFS-14) |  | Generic |
| 2^43, 98^ | Hidradenitis Suppurativa Quality of Life (HiSQOL) |  | Hidradenitis suppurativa |
| 1^169^ | Hidradenitis Suppurativa Quality of Life-24 (HSQoL-24) |  | Hidradenitis suppurativa |
| 1^118^ | HIDRAdisk |  | Hidradenitis suppurativa |
| 1^26^ | HidroQoL |  | Hyperhidrosis |
| 1^53^ | Hospital Anxiety Depression Scale (HADS) |  | Generic |
| 1^47^ | ICECAP-A |  | Generic |
| 1^41^ | Ichthyosis-specific measure of quality of life (IQoL-32) |  | Ichthyosis |
| 1^77^ | Individual Burden of Psoriasis (I-BOP) |  | Psoriasis |
| 1^136^ | Internalized Stigma Scale (ISS) |  | Generic |
| 1^68^ | Inverse Psoriasis Burden of Disease (IPBOD) |  | Psoriasis |
| 1^144^ | ItchyQoL |  | Pruritus |
| 1^149^ | Liebowitz Social Anxiety Scale (LSAS) |  | Generic |
| 1^55^ | Melasma Quality of Life scale (MELASQOL) |  | Melasma |
| 2^55, 103^ | Melasma quality of life scale (MELASQoL) Brazilian Portuguese |  | Melasma |
| 1^138^ | Multidimensional Assessment of Interoceptive (bodily sensations) Awareness-2 (MAIA-2) |  | Generic |
| 1^147^ | Nail psoriasis quality of life scale (NPQ10) |  | Nail psoriasis |
| 1^116^ | Occupational Contact Dermatitis Disease Severity Index (ODDI) |  | Contact dermatitis |
| 1^87^ | Patient Generated Index (PGI) |  | Generic |
| 1^42^ | Patient global assessment (PtGA) for hidradenitis suppurativa |  | Hidradenitis suppurativa |
| 1^33^ | Patient Health Questionnaire-9 (PHQ9) |  | Generic |
| 1^78^ | Patient Unique Stigmatization Holistic tool in dermatology (^78^) |  | Generic |
| 1^31^ | Patient-Reported Outcomes Measurement Information System Global Health (PGH) |  | Generic |
| 1^59^ | Person-Centred Dermatology Self-care Index (PeDeSI) |  | General dermatological |
| 3^69, 80, 98^ | Pictorial representation of illness and self measure (PRISM) |  | Generic |
| 1^32^ | PROMIS Itch Questionnaire Mood and Sleep (PIQ-MS) |  | Pruritus |
| 1^119^ | Prurigo Activity Score (PAS) |  | Prurigo nodularis |
| 2^49, 70^ | PSOdisk |  | Psoriasis |
| 1^25^ | PSO-LIFE |  | Psoriasis |
| 1^85^ | Psoriasis Disability Index (PDI) Chinese |  | Psoriasis |
| 1^101^ | Psoriasis Disability Index (PDI) Sinhala version |  | Psoriasis |
| 1^75^ | Psoriasis Family Index-15 (PFI-15) |  | Psoriasis |
| 1^105^ | Psoriasis Index of Quality of Life (PSORIQoL) |  | Psoriasis |
| 1^35^ | Psoriasis Symptom Diary |  | Psoriasis |
| 1^48^ | Psoriasis Symptom Scale (PSS) |  | Psoriasis |
| 1^37^ | Psoriasis Symptoms and Impacts Measure (P-SIM) |  | Psoriasis |
| 1^58^ | Psychosomatic Scale for Atopic Dermatitis |  | Atopic dermatitis |
| 1^66^ | Quality of life evaluation in epidermolysis bullosa (QoLEB) Brasilian Portuguese |  | Epidermolysis bullosa |
| 1^110^ | The Quality of Life in Hand Eczema Questionnaire (QOLHEQ) Japanese |  | Eczema/Hand eczema |
| 1^29^ | Quality of Life in Hand Eczema Questionnaire (QOLHEQ) Dutch |  | Eczema/Hand eczema |
| 1^140^ | Quality of Life Index for Atopic Dermatitis (QoLIAD) |  | Atopic dermatitis |
| 1^82^ | REFlective evaLuation of psoriasis Efficacy of Treatment and Severity (REFLETS) QoL |  | Psoriasis |
| 1^71^ | Rosacea-specific Quality-of-Life instrument (RosQol) |  | Rosacea |
| 1^27^ | Sarcoidosis Assessment Tool (SAT) |  | Sarcoidosis |
| 1^92^ | Severity and Area Score for Hidradenitis (SASH) |  | Hidradenitis suppurativa |
| 1^123^ | Skin Cancer Index |  | Cancer |
| 1^62^ | Skin Cancer Quality of Life Impact Tool (SCQOLIT) |  | Cancer |
| 1^36^ | Skin Cancer Quality of Life questionnaire (SCQoL) |  | Cancer |
| 1^142^ | Skin Picking Disorder (SPD, Brazilian) |  | Skin picking disorder |
| 1^89^ | Skindex -17 Brazil |  | General dermatological |
| 5^63, 81, 84, 94, 107^ | Skindex-16 Brazilian (2), English (2), Chinese |  | General dermatological |
| 1^84^ | Skindex-29 Chinese |  | General dermatological |
| 1^109^ | Skindex-29 Serbian |  | General dermatological |
| 2^114, 115^ | Uraemic Pruritus in Dialysis Patients (UP-Dial) |  | Uraemic Pruritus |
| 2^97, 170^ | Vitiligo Impact Scale (VIS) |  | Vitiligo |
| 1^145^ | Vitiligo specific quality of life instrument (VitiQoL) Chinese |  | Vitiligo |
| 1^100^ | Vitiligo-specific health-related quality of life instrument (VitiQol) |  | Vitiligo |
| 1^88^ | Vitiligo-specific health-related quality of life instrument (VITIQoL) Polish |  | Vitiligo |
| 2^60, 65^ | Vitiligo-specific health-related quality of life instrument (VitiQoL) Portuguese Brazilian |  | Vitiligo |
| 1^129^ | Vitiligo Treatment Impact score (VITs) |  | Vitiligo |
| 1^127^ | Vitiligo Life Quality Index (VLQI) |  | Vitiligo |
| 1^102^ | Vulval Intraepithelial Neoplasia (VIN) questionnaire |  | Vulval intraepithelial neoplasia |
| 1^125^ | Vulvar Quality of Life Index (VQLI) |  | Vulvar disease |
| 1^128^ | Women's Androgenetic Alopecia Quality of Life Questionnaire (WAA-QoL) Brazilian Portuguese |  | Alopecia |
| 1^76^ | World Health Organization Quality of Life Short Version (WHOQOL-BREF) |  | Generic |
| 1^113^ | QLQ C30 European Organization for Research and Treatment of Cancer (EORTC) core QL Questionnaire - Cancer (QLQ-C30) |  | Cancer |
| 1^131^ | Short form 12 (SF-12) |  | Generic |
